# Preferential binding of unsaturated hydrocarbons in aryl-bisimidazolium·cucurbit[8]uril complexes furbishes evidence for small-molecule π–π interactions†
†Electronic supplementary information (ESI) available: Materials and methods section, fluorescence titrations, ITC experiments, recognition of diiodine, and additional NMR data. See DOI: 10.1039/c9sc03282g


**DOI:** 10.1039/c9sc03282g

**Published:** 2019-10-17

**Authors:** Steven J. Barrow, Khaleel I. Assaf, Aniello Palma, Werner M. Nau, Oren A. Scherman

**Affiliations:** a Melville Laboratory for Polymer Synthesis , Department of Chemistry , University of Cambridge , Lensfield Road , Cambridge , CB2 1EW , UK . Email: oas23@cam.ac.uk; b Department of Life Sciences and Chemistry , Jacobs University Bremen , Campus Ring 1 , D-28759 Bremen , Germany . Email: w.nau@jacobs-university.de

## Abstract

Whilst cucurbit[*n*]urils (CB*n*) have been utilized in gas encapsulation, only the smaller CB*n* (*n* = 5 and 6) have utility given their small cavity size. In this work, we demonstrate that the large cavity of CB8 can be tailored for gaseous and volatile hydrocarbon encapsulation by restricting its internal cavity size with auxiliary aryl-bisimidazolium (Bis, aryl = phenyl, naphthyl, and biphenyl) guests. The binding constants for light hydrocarbons (C ≤ 4) are similar to those measured with CB6, while larger values are obtained with Bis·CB8 for larger guests. A clear propensity for higher affinities of alkenes relative to alkanes is observed, most pronounced with the largest delocalized naphthalene residue in the auxiliary Bis guest, which provides unique evidence for sizable small-molecule π–π interactions.

## Abstract 2

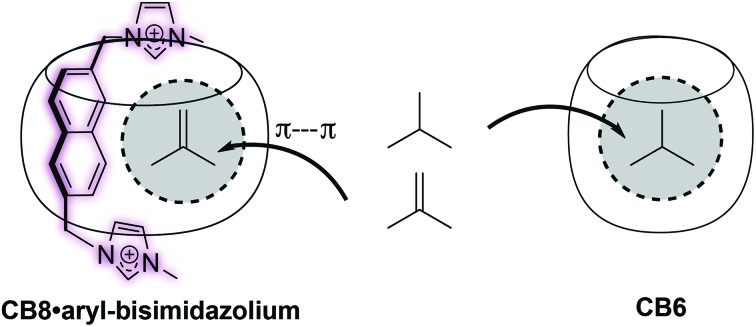
Restricting the internal cavity size of cucurbit[8]uril with auxiliary guests sets up an intermolecular interaction chamber for hydrocarbons, which provides insights into dispersion, arene–hydrocarbon interactions, and desolvation effects.

## Introduction

The development of refined gas-encapsulation materials is driven by economic (methane binding) and environmental (CO_2_ capture) promises and holds additional potential for advanced sensing and photochemical applications.^1^,^2^ Besides metal–organic frameworks,^3^–^5^ porous coordination polymers^6^,^7^ exhibit very high surface areas and have demonstrated selectivity and capacity for adsorbing gases like CO_2_, making them competitive candidates for gas encapsulation, with the common disadvantage of being water sensitive.^8^–^10^ Discrete host–guest chemistry presents an alternative approach largely by-passing stability issues.^11^–^13^ In particular, cucurbit[*n*]urils, CB*n*, present a class of macrocycles that have demonstrated gas uptake capacities comparable to several porous materials.^11^,^12^,^14^–^21^ CB*n* are based on glycoluril subunits, which have been shown to encapsulate a variety of cationic and neutral guest species.^20^–^27^ The unique binding capabilities of the CB*n* family arise due to ion–dipole interactions at the carbonyl-lined portals, in addition to the size and hydrophobicity of the inner cavity.^28^ The size of the inner cavity of CB*n* is a powerful predictor in terms of the breadth of chemical species that can bind to the macrocycle. CB8 can bind two small aromatic compounds simultaneously, whereas the smaller CB7 and CB6 can generally bind only one at a time.^23^ As a consequence of size complementarity, gas binding tends to be favoured by the smaller CB5 and CB6 homologues.^14^,^21^,^29^–^31^

**Fig. 1:**
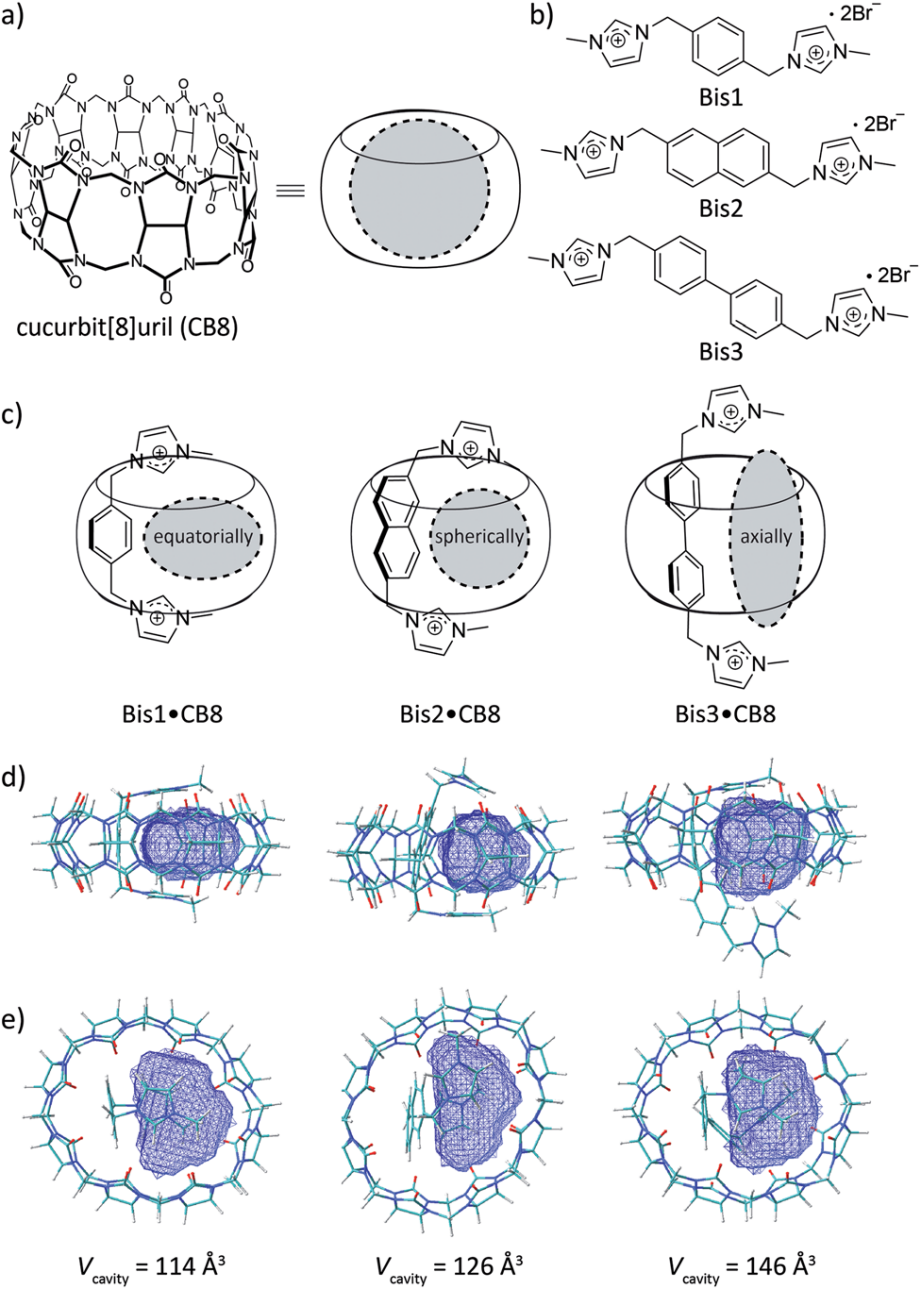
Chemical structures of (a) CB8, (b) the bisimidazolium auxiliary guests, and (c) their schematic complexes with CB8; differently shaped cavities shown in grey. (d) and (e) show the side and top views of the DFT-optimized (wB97XD/3-21G* level of theory) binary complex structures with the respective accessible cavity volume (*V*_cavity_) highlighted in blue.

Herein, we demonstrate that aryl-bisimidazolium (Bis) guests can tailor the interior cavity space of CB8 toward preferential binding of gaseous and volatile hydrocarbons (Fig. 1) with increased selectivity for unsaturated ones. The Bis guests differ based on the hydrophobic linker between the two imidazolium units, specifically, phenyl (Bis1), naphthyl (Bis2), and biphenyl (Bis3). We show that a wide variety of hydrocarbon guests can be encapsulated, *via*^1^H NMR and fluorescence spectroscopy, and that by changing the hydrophobic moiety within the Bis guests, CB8 can be made selective toward particular guest molecules by changing the size and shape of the remaining cavity space within the macrocycle. The three Bis guests^32^ (Fig. 1) have been previously used in conjunction with CB8 to enable encapsulation of small solvent molecules in a 1 : 1 : 1 binding stoichiometry and other auxiliary guests without “lids”.^33^,^34^

Auxiliary aromatic guest–hydrocarbon complexes have been earlier assembled inside capsular assemblies or coordination cages.^35^,^36^ How these auxiliary guests allow for a large macrocycle to encapsulate small molecular species lies in the fact that the auxiliary guests occupy a large portion of the internal cavity, such as that of CB8, effectively altering the packing coefficient (PC) of small molecules inside the cavity.^28^,^37^ Fig. 1d and e show DFT calculations that reveal the extent of the internal cavity volume restriction for a U-shaped conformation of the Bis guests in CB8 that templates the encapsulation of a second guest. Vacant CB8 has a cavity volume of 367 Å^3^,^28^ however, the formation of Bis1·CB8, Bis2·CB8, and Bis3·CB8 complexes reduces the available cavity volume to 114, 126, and 146 Å^3^, respectively (Table 1). In terms of capacity, these complexes fall in between CB5 and CB6 for which gas encapsulation has been documented,^11^,^16^,^17^,^19^,^20^,^29^,^31^ driven, among others, by the release of high-energy water (for CB6)^38^,^39^ and cavitation energy (for CB5).^21^ Moreover, as reflected by the calculations, the resulting templated cavities differ in shape, from equatorially elongated (for Bis1·CB8) to spherical (for Bis2·CB8) to axially elongated (for Bis3·CB8), which offers an interesting design approach. Finally, by varying the size of the aryl unit from phenyl to naphthyl and biphenyl, it should not only be possible to vary size and shape of the resulting cavity, but also secondary C–H–π and π–π guest–guest interactions, the importance of which remains under debate.^33^,^36^,^40^–^51^ In addition to playing a role in rational secluded-cavity design, the Bis guests are fluorescent, which enables working at micromolar concentrations and offers the opportunity for direct and real-time optical sensing of gaseous and volatile guests at micromolar concentrations.^19^,^20^

**Table 1:** Calculated cavity volumes of CB*n* and Bis·CB8 complexes

CB*n*	Cavity volume/Å^3^	Bis·CB8	Cavity volume/Å^3^
CB5	68^*a*^	Bis1·CB8	114^*b*^
CB6	142^*a*^	Bis2·CB8	126^*b*^
CB7	242^*a*^	Bis3·CB8	146^*b*^
CB8	367^*a*^		

^*a*^From ref. 28.

^*b*^Calculated from optimized structures, see Fig. 1.

## Results and discussion

The formation of 1 : 1 host–guest complexes between CB8 and Bis1–Bis3 with high binding constants (*K*_a_ > 10^6^ M^–1^) has been established by using different spectroscopic methods (see ESI,† and ref. 32). As anticipated, the binary Bis·CB8 complexes should act as receptors for small molecules, which would otherwise not complex to free CB8. Visual evidence for the entrapment of small molecules – and for their potential in sensing – can be obtained by their addition to diiodine (I_2_) solutions, which affects an immediate color change (Fig. S11†) from yellow-brown (in water) to violet, an iodine color otherwise only observed in nonpolar solvents. In this case, the binding constants were determined by direct UV-visible titrations (*K*_a_ ∼ 2 × 10^4^ M^–1^, see Table 2 and Fig. S11†). These binding constants are lower than the previously measured value with CB6 (*K*_a_ = 1.4 × 10^6^ M^–1^)^52^ but similar to that obtained for α-cyclodextrin.^53^ Accordingly, Bis·CB8 complexes are competitive binders for small guests.

**Table 2:** Association constants (*K*_a_) of hydrocarbons with Bis·CB8 systems and CB*n*, measured in neat water

Guest	*K*_a_ ^*a*^/(10^3^ M^–1^)				
	Bis1·CB8	Bis2·CB8	Bis3·CB8	CB6^*b*^	CB7^*c*^
Methane	0.6 ± 0.1	0.4 ± 0.1	0.6	<2	3
Ethane		0.5 ± 0.1		24	3.4
Propane		14		180	6
*n*-Butane	580	89	35 ± 11	280	170
*cis*-Butene		430		150	35
*trans*-Butene		24		21	14
Isobutane	186	31	410	850	265
Isobutene	18	65	66	84	43
Neopentane	14	5600	9.3	<2	1000
Cyclopentane		67		1300	196
Cyclopentene	290^*d*^	260 [480]^*d*^	960^*d*^	140^*e*^	25^*e*^
Cyclopentanol	2.0^*d*^	6.7^*d*^	5.9^*d*^		
Cyclohexane		66		<2	1500
1,3-Cyclohexadiene	88^*d*^	530^*d*^	1900^*d*^		
Benzene	85^*d*^	170 [520]^*d*^	[710]^*d*^	<2	17
Phenol	3.0^*d*^	32 ± 5^*d*^	18^*d*^		
I_2_	17 ± 2^*e*^	21 ± 8^*f*^	19 ± 4^*e*^	1400^*g*^	100^*g*^

^*a*^Error in *K*_a_ values is 15% unless stated differently.

^*b*^From ref. 20.

^*c*^From ref. 19.

^*d*^Values in square brackets measured by ITC, 10% error unless stated differently, see Table S3 in ESI.

^*e*^Measured in this work by indicator displacement.

^*f*^Measured by UV-vis absorption titrations, see ESI.

^*g*^From ref. 52.

**Fig. 2:**
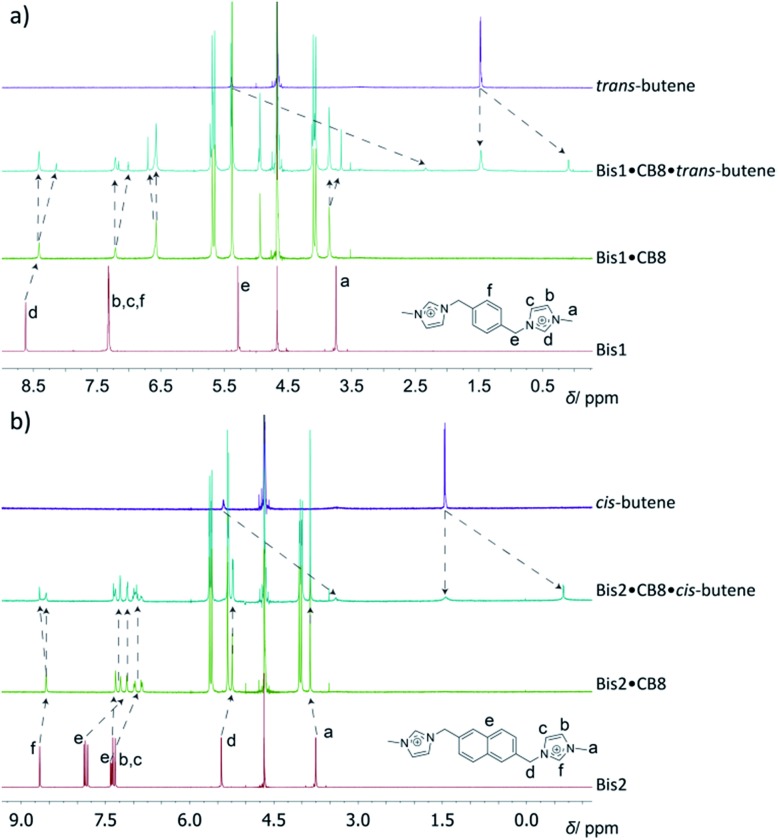
^1^H NMR spectra for the binding of (a) *trans*-butene to the Bis1·CB8 complex and (b) *cis*-butene to Bis2·CB8, in D_2_O.

For optically inactive guests, such as hydrocarbons, ^1^H NMR spectroscopy was used for structural characterization of the ternary hydrocarbon complexes; very large upfield shifts (>2 ppm) were observed for the encapsulated guests (see Fig. 2 and ESI†), which were found to be in slow exchange even for the smallest guests. This was in contrast to the binary Bis·CB8 complexes, in which host and guest were in fast exchange, resulting in sharper NMR bands. For example, in the ^1^H NMR spectra for complex formation of the Bis1·CB8·methane system (see ESI†), significant shifts and line broadening were observed. While upfield shifts up to 1 ppm are characteristic for CB*n* encapsulation itself,^19^ the larger values in the Bis1·CB8 guest complexes are due to an anisotropic shielding effects from the adjacent aryl groups of the pre-complexed Bis guests.^8^ The ^1^H NMR spectra for the formation of the *trans*-butene·Bis1·CB8 complex can be seen in Fig. 2a. An upfield shift of the peaks associated with *trans*-butene of 1.4–3.0 ppm is observed once the guest is encapsulated within the Bis1·CB8 complex. Similarly, the encapsulation of *cis*-butene inside the Bis2·CB8 complex was confirmed by the upfield shifts of the guest peaks (Fig. 2b). Other gases, including CO_2_ and SF_6_, also form complexes with the Bis·CB8 systems (see ESI†). The complexation of SF_6_ with the Bis·CB8 was investigated by using ^19^F NMR, in which the fluorine atoms experienced an upfield shift (see Fig. S20†), in accordance with the inclusion of perfluorinated guests within CB*n*.^26^

**Fig. 3:**
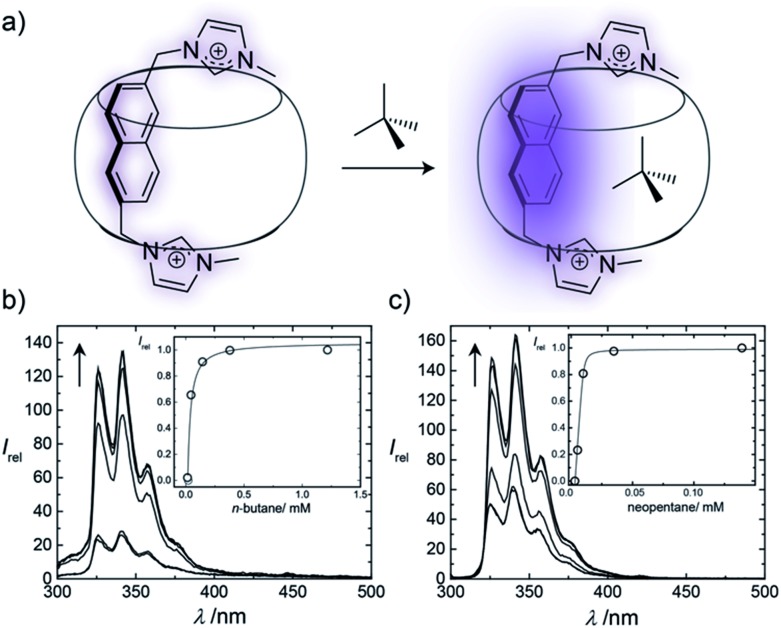
(a) The encapsulation of a second guest within a Bis·CB8 complex enhances the fluorescence of the first guest; changes in fluorescence can be directly correlated to the binding strength of the second guest. (b) and (c) Changes in fluorescence spectra for Bis2·CB8 complexes, plotted *versus* wavelength and second guest concentration (inset) for (b) *n*-butane and (c) neopentane.

The binding affinities of selected hydrocarbons were independently measured by using fluorescence titrations, through monitoring the fluorescence response of the auxiliary Bis guests upon binding of the second guest molecule (Fig. 3 and ESI†). For gases, pressure was adjusted to control their concentration (see ESI†), while for volatile liquid guests stock solutions were used. Although concentration variations in gas titrations are greatly limited compared to conventional titrations with stock solutions,^19^,^20^ aqueous hydrocarbon solubilities are accurately known, which allowed for good reproducibilities. Enhancement of the fluorescence intensity was observed upon formation of the 1 : 1 : 1 ternary complexes, which is attributed to the replacement of the residual water molecules from the cavity by the hydrophobic guests (Fig. 3). As can be seen from Fig. 3 and Table S2,† the different hydrocarbons showed markedly different fluorescence responses. The measured binding affinities are shown in Table 2, as well as data for CB6 (ref. 20) and CB7 (ref. 19) (the PC analysis for each system is provided in Table S1†).

The binding constants vary with the auxiliary guest. For example, *n*-butane binds more strongly to the Bis1·CB8 complex compared to Bis2·CB8 and Bis3·CB8, while neopentane binds tightly to Bis2·CB8. This might be attributable to the different cavity shapes (Fig. 1), in which the more spherical cavity of the Bis2·CB8 complex prefers globular guests such as neopentane, while those of Bis1·CB8 and Bis3·CB8 preferentially bind elongated guests, along the equatorial and axial voids, respectively. Interestingly, Bis2·CB8 markedly and consistently showed higher binding affinities for alkenes than for the corresponding alkanes. Specifically, isobutene and cyclopentene bind more strongly than isobutane and cyclopentane, respectively. The opposite selectivity applies for CB6 and CB7, to which saturated hydrocarbons bind more strongly than their unsaturated counterparts.^19^,^20^ Strong binding of alkenes is counterintuitive, because they are 3–5 times more water soluble (Table 3) than alkanes and, therefore, less hydrophobic. This hints at another prevailing aspect that contributes to hydrocarbon binding in these Bis·CB8 complexes, namely, π–π interactions between the first guest and the second guest. Although the estimated cavity size of Bis2·CB8 is slightly less than that of CB6 (Table 1), it binds cyclohexane better than CB6 (6.6 × 10^4^*versus* <0.2 × 10^4^ M^–1^), presumably because the ternary complex is somewhat more flexible and can adapt its lids to the encapsulated guest. However, it binds cyclohexane less tightly than CB7 (6.6 × 10^4^*versus* 150 × 10^4^ M^–1^), which can be attributed in this case to a tight packing (PC = 81% *versus* 72%). In contrast, benzene binds to the Bis2·CB8 system 10-times more strongly than to CB7.

**Table 3:** Guest solubility (*S*), guest volume (*V*), polarizability (*α*), hydration free energy (Δ*G*_hydr_), binding free energy (Δ*G*_a_) as measured in neat water, and corrected binding free energy 
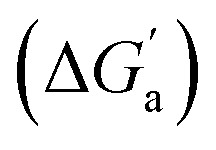
 for different CB*n* host–guest complexes; all energy values in kcal mol^–1^

Guest	*S* ^*a*^/mM	*V* ^*b*^/Å^3^	*α* ^*c*^/Å^3^	Δ*G*_hydr_^*d*^	Bis2·CB8		CB6		CB7	
					Δ*G*_a_^*e*^	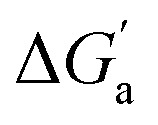 ^*f*^	Δ*G*_a_^*g*^	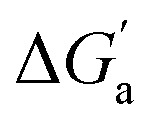 ^*f*^	Δ*G*_a_^*h*^	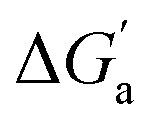 ^*f*^
Methane	1.40	29	2.59	1.99	–3.53 ± 0.17	–1.54			–4.74	–2.75
Ethane	1.89	45	4.43	1.82	–3.73 ± 0.13	–1.92	–5.98	–4.16	–4.82	–3.00
Propane	1.52	63	6.37	1.94	–5.66	–3.72	–7.17	–5.23	–5.15	–3.21
*n*-Butane	1.25	80	8.2	2.06	–6.75	–4.69	–7.43	–5.37	–7.14	–5.08
*cis*-Butene	3.99	74	8.0	1.37	–7.69	–6.31	–7.06	–5.69	–6.20	–4.83
*trans*-Butene	4.11	74	8.49	1.36	–5.98	–4.62	–5.90	–4.54	–5.66	–4.30
Isobutane	0.92	79	8.14	2.24	–6.13	–3.89	–8.09	–5.85	–7.40	–5.16
Isobutene	4.69	75	8.29	1.28	–6.57	–5.29	–6.72	–5.44	–6.32	–5.04
Neopentane	0.46	96	9.99	2.65	–9.21	–6.56			–8.19	–5.54
Cyclopentane	2.24	86	9.15	1.20	–6.58	–5.38	–8.33	–7.13	–7.22	–6.02
Cyclopentene	7.93	81	8.87^*i*^	0.55	–7.39	–6.84	–7.02	–6.47	–6.00	–5.45
Cyclohexane	0.69	102	11.0	1.19	–6.57	–5.38			–8.4	–7.21
Benzene	22.79	89	10.7	–0.89	–7.13	–8.02			–5.77	–6.66
I_2_	0.13^*i*^	71	10.3^*j*^	–1.20	–5.90 ± 0.28	–7.10	–8.38^*k*^	–9.58	–6.82^*k*^	–8.02

^*a*^From ref. 62.

^*b*^Obtained from AM1-optimized structures by using the QSAR module of Hyperchem.

^*c*^From ref. 19.

^*d*^Calculated from the solubility (*S*) and vapor pressure (*p*_vap_) according to Δ*G*_hydr_ = –*RT* ln(*Sp*^0^/*p*_vap_) – 1.90 kcal mol^–1^ with *p*^0^ = 101.325 kPa and *p*_vap_ in kPa.

^*e*^Obtained from binding constants in Table 2; error ±0.10 kcal mol^–1^, unless explicitly stated.

^*f*^ 
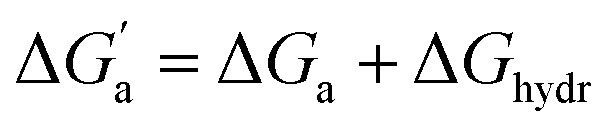
.

^*g*^From ref. 20.

^*h*^From ref. 19.

^*i*^From ref. 63.

^*j*^From ref. 64.

^*k*^From ref. 52.

To corroborate the preferential binding of unsaturated over saturated guests with a different method, we conducted isothermal titration calorimetry (ITC) experiments, which afforded additional thermochemical information (see ESI, Table S3 and Fig. S10†). This technique requires higher concentrations of the guest, which limited the accessible guest range; to remedy, we included two additional water-soluble derivatives (phenol and cyclopentanol), which set up an additional pair to probe for π–π interactions. In general, guest binding to the Bis·CB8 binary complexes was found to be enthalpically driven by 8–12 kcal mol^–1^, with a negative entropic component (Fig. S10†), a signature which could be related to the non-classical hydrophobic effect (removal of high-energy water molecules from the cavity).^38^,^39^,^54^ The binding constants for cyclopentene and benzene with Bis2·CB8 obtained by ITC and fluorescence displacement (see Table 2) showed satisfactory agreement, if one considers the known systematic variations in binding constants when different methods are being employed.^23^ The higher binding affinity for phenol than cyclopentanol with the three Bis·CB8 systems confirmed π–π interactions: although both guests have identical volume^55^ and although the highly water-soluble phenol should display a lower hydrophobic component to the driving force, it has a higher affinity to the Bis·CB8 receptors than the less water-soluble cyclopentanol (see also Table S4 in ESI†). This affinity pattern for the Bis·CB8 receptors (guests with phenyl residue binding more strongly than those with cyclopentyl ones) contrasts that observed early for CB6 (three order of magnitude lower binding of phenyl than of the corresponding cyclopentyl guests)^20^,^55^ and later for CB7.^19^

**Fig. 4:**
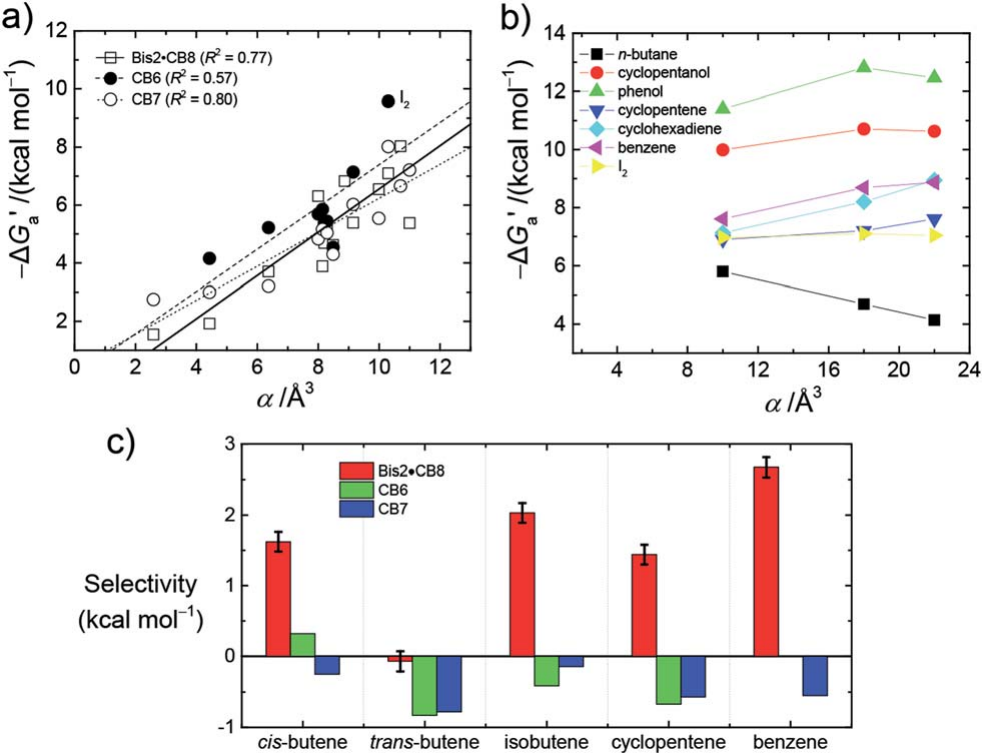
(a) Plot of 
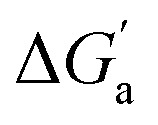
 as a function of guest polarizability (*α*) for Bis2·CB8, CB6, and CB7. (b) Plot of 
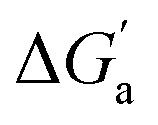
 *versus α* of aryl-spacer in the Bis systems;^58^*α* calculated at the B3LYP/aug-ccpvdz level of theory, see ESI.† (c) Bar graph visualizing the selectivity of different CB cavities towards unsaturated hydrocarbons *versus* their fully saturated counterparts, with 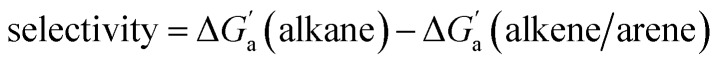; a positive value indicates stronger binding of the particular alkene/arene, a negative one a preference for the alkane.

The hydrocarbons in Table 2 cover a homologous series with a wide range of guest size and hydrophobicity, but without interference from electrostatic interactions (ion–ion, ion–dipole, dipole–dipole, and hydrogen-bonding).^19^ In order to decouple the classical hydrophobic effect^38^,^39^ associated with guest desolvation as a driving force for host–guest complexation, the hydration free energy of the guest – experimentally known for hydrocarbons – needs to be added to the experimental binding free energy.^19^,^33^ This affords a guest-desolvation corrected value for the driving force 
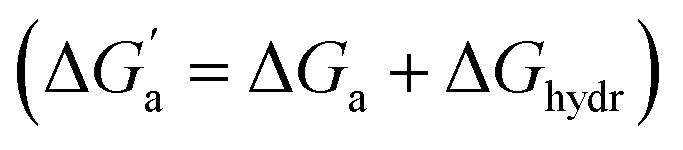
 that reflects the intrinsic affinity of a particular hydrocarbon to Bis2·CB8, CB6, and CB7 (Table 3). For each host, the 
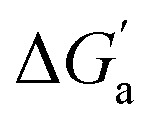
 values increase roughly with the size/polarizability of the hydrocarbons (Table 3 and Fig. 4). This trend is reasonable as dispersion interactions^56^,^57^ are expected to increase with guest size^19^,^21^ as long as the PC of a guest does not become too large.^24^,^28^,^58^,^59^

A comparison of guest desolvation-corrected free binding energies 
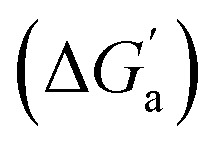
 of the arene-loaded Bis·CB8 complexes with the equally large CB6 cavity is immediately instructive. This is because the interpretation of the driving force can be reduced to (i) direct host–guest interactions (dispersion, π–π, C–H–π, and cation–π interactions) and (ii) the non-classical hydrophobic effect (removal of high-energy water).^38^,^39^ Considering first the alkanes (and iodine), the 
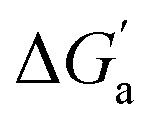
 values for CB6 are consistently higher (more negative) than the corresponding ones for Bis2·CB8. As both the presence of C–H–π interactions (alkane–Bis) and the higher efficiency of dispersion interactions would actually predict a higher affinity for Bis2·CB8, this trend must be traced back to the non-classical hydrophobic effect, which is known to be larger in neat CB*n* cavities than in aromatically laced ones, such as the cavities of calixarenes and pillararenes.^39^,^60^,^61^

Through the variation of the aryl group of the Bis guests, the size/polarizability of the aromatic surfaces can be systematically varied from benzyl (*α* = 10 Å^3^) to naphthyl (*α* = 18 Å^3^) to biphenyl (*α* = 22 Å^3^). Except for the flexible *n*-butane and isobutane, the 
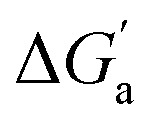
 values of the guests were found to increase from Bis1 (phenyl) to Bis2 (naphthyl) by ∼1 kcal mol^–1^, but not upon going from Bis2 to Bis3 (Fig. 4b). Presumably, although Bis3 has a higher polarizability, the nonplanar, twisted geometry of the biphenyl (Bis 3) system (see Fig. 1d and e) introduces more stringent steric requirements.

Most striking was the variation of the 
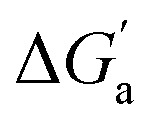
 values between unsaturated and saturated hydrocarbons for Bis2·CB8 in comparison to CB6 and CB7. We defined an intrinsic selectivity, 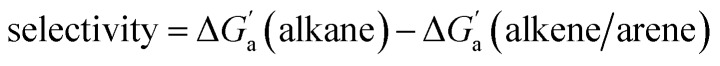, which reports on the relative stabilization of a π system *versus* the hydrocarbon reference (Fig. 4c). Even if one disregards steric hindrance effects for specific guests,^58^ it transpires that the non-lined CB6 and CB7 cavities favor binding of the saturated hydrocarbon analogues by *ca.* 0.5 kcal mol^–1^ (green and blue bars). This can be accounted for with increased dispersion interactions of the (larger) alkanes *versus* their unsaturated counterparts inside the CB cavities.^19^ The naphthyl auxiliary induced a reversal in selectivity: Bis2·CB8 favors the binding of the unsaturated hydrocarbons by *ca.* 1.5 kcal mol^–1^ (red bars). Because desolvation effects have been corrected for in the 
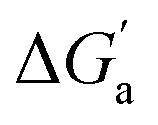
 values or should remain constant (removal of cavity water from the same cavity), the preferential binding of alkenes and arenes by Bis2·CB8 must be attributed to additional stabilizing intermolecular interactions that are specific for unsaturated hydrocarbons and that become particularly large for aromatic guests (benzene). These conclusions remain robust when larger method-to-method variations in absolute binding constants (ITC instead of fluorescence displacement titrations) are considered (Tables 3 and S4 in ESI†).

We assign these additional interactions as π–π interactions, in line with what chemical intuition predicts but what has been notoriously difficult to experimentally verify until now.^33^,^43^–^49^,^65^ The stabilizing π–π interactions in the alkene/arene Bis2·CB8 complexes must be sufficiently large (*ca.* 2 kcal mol^–1^) to even overwhelm the counteracting (*ca.* –0.5 kcal mol^–1^) dispersion interactions. From a molecular recognition point of view, CBs are prototypal hosts for saturated hydrocarbons, but can be converted, *e.g.*, by lining with aromatic guests in Bis2·CB8, into prime receptors for unsaturated hydrocarbons (and for the spherical neopentane). Interestingly, Bis1 and Bis3 do not display comparable magnitudes of π–π interactions, as reflected in the lower binding of isobutene compared to isobutane, which could be accounted for by the smaller phenyl (Bis1) or twisted biphenyl (Bis3) π systems of these two auxiliary guests. A related study by Masson,^33^ in which ternary hydrocarbon complexes had also been formed with a phenyl group as auxiliary, did not afford any evidence for π–π interactions with alkenes and arenes; instead, CH–π interactions were assigned as a dominant driving force. Studies with CB8 containing a (biphenyl-related) methyl viologen as auxiliary guest and aromatic guests have not afforded evidence for charge transfer (π–π stacking) interactions as driving force for ternary complex formation either.^46^ Presumably, the larger naphthalene π system in Bis2 is crucial to produce thermochemically significant effects.

We also conducted quantum-chemical calculations to theoretically evaluate the preferred co-conformations in the Bis2·guest complexes (see ESI†). We tested the B3LYP level of theory as a starting point, the wB97XD level to include dispersion interactions,^19^ and also the M06-2X level recently recommended for alkene–arene π–π stacking interactions,^66^ all at a common 6-31+G* basis set. We selected three unsaturated guests (*cis*-butene, isobutene, and benzene) and optimized their geometries in two opposing co-conformations, one which would allow for π–π stacking with the naphthalene unit and an approximately orthogonal one which would lead to a C–H–π orientation with the naphthalene ring (see Fig. S50 in ESI†). The calculations were reaffirming in terms of the proposed size fitting in the structures of the ternary complexes (Fig. 1) and produced, regardless of the selected method and guest, the π–π stacked co-conformations as the energetically preferred geometries (see Table S5 in ESI†).

Although the original goal in our study was directed towards selective binding of hydrocarbons, it turned out that Bis·CB8 systems can be used as alternatives to classical molecular balances^67^–^73^ in order to evaluate intermolecular interactions, providing insights into the interplay between desolvation effects and direct molecular interactions, including dispersion and π–π interactions.^67^–^69^,^72^ The Bis·CB8 systems provide a solvent-shielded environment through the CB walls, while the imidazolium moieties act as ‘lids’ that close the CB carbonyl portals. To further expand the usage of Bis·CB8 systems as “intermolecular interaction chambers”, see Fig. 3a, we plan to study alkyl, perfluoroalkyl, and substituted aryl linkers between the imidazolium units. It may well be that intermolecular interactions inside the chambers become more pronounced than in the corresponding molecular balances, due to more effective desolvation and forced proximity.

## Conclusions

In summary, we have shown that CB8, one of the largest members of the CB*n* family, can be tailored towards selective hydrocarbon binding. This was achieved through the formation of aryl-bisimidazolium complexes, the purpose of which was three-fold. Firstly, the aromatic units are fluorescent, such that binding events can be directly monitored. Secondly, the auxiliaries restrict the available cavity space within CB8, which increases the packing coefficient and favours the complexation of small guests, including gases. Thirdly, the incorporation of a naphthyl unit between the imidazolium caps allows for sizable π–π interactions with the encapsulated small guest molecules, which tips the selectivity towards alkenes and arenes.

## Conflicts of interest

There are no conflicts to declare.

## Supplementary Material

Supplementary information
