# Supplementary material for: Preferential binding of unsaturated hydrocarbons in aryl-bisimidazolium·cucurbit[8]uril complexes furbishes evidence for small-molecule π–π interactions
Source: Chem Sci. 2019 Oct 17;10(44):10240–6. doi: 10.1039/c9sc03282g (PMC7006508; doi:10.1039/c9sc03282g)
Supplement: Supplementary file 1 [file SC-010-C9SC03282G-s001.pdf]

Electronic Supplementary Material (ESI) for Chemical Science.
This journal is © The Royal Society of Chemistry 2019

Electronic Supplementary Information

Preferential Binding of Unsaturated Hydrocarbons in Aryl–Bisimidazolium•Cucurbit[8]uril Complexes Furbishes Evidence for Small-Molecule π–π Interactions

Steven J. Barrow,[a] Khaleel I. Assaf,[b] Aniello Palma,[a] Werner M. Nau,[b] Oren A. Scherman[a]

[a] *Melville Laboratory for Polymer Synthesis, Department of Chemistry, University of Cambridge, Lensfield Road, Cambridge, CB2 1EW, U.K*

[b] *Department of Life Sciences and Chemistry, Jacobs University Bremen, Campus Ring 1, D-28759 Bremen, Germany*

Table of Contents

1. Materials and Methods 2
2. Packing Coefficient Analysis 3
3. Fluorescence Binding Titrations 4
4. Isothermal Titration Calorimetry Experiments 8
5. Diiodine Binding to the Bis•CB8 11
6. NMR Studies 12
7. DFT Calculations 31
8. References 33

1. Materials and Methods

Gases of highest commercial purity (≥ 99%) were purchased from Air Liquide, Germany, except for *cis*- and *trans*-butene (Sigma-Aldrich, Germany) and neopentane (ChemSampCo, USA). Liquid hydrocarbons were from Sigma-Aldrich, Germany. UV-vis measurements were performed on a Varian Cary 4000 UV-Visible spectrophotometer and fluorescence measurements were done on a Varian Cary Eclipse fluorimeter.

Fluorescence titrations at different gas concentrations (pressures) were performed in custom-made degassable quartz cuvettes attached to a Schlenk line. Our experimental set-up allowed us to perform the fluorescence titrations of the degassed Bis•CB8 solutions (three freeze-pump-thaw cycles), at atmospheric pressure of the hydrocarbon gas, and at 3-4 successively reduced pressures (as adjusted by fixed volume expansions from the reservoir into the cuvette volume) until the partial pressure of the gas fell below the vapor pressure of water (which needed to be subtracted from the measured pressure). Since the aqueous hydrocarbon solubilities are accurately known and can be related to pressure by Henry's law, titrations with good reproducibility resulted even if the accessible data points were restricted to 5-6 equal to and below atmospheric pressure.

^{1}H NMR spectra were recorded on a JEOL ECX 400 MHz NMR spectrometer. Quantum-chemical calculations were performed with the Gaussian 09 package, utilizing density functional theory (DFT) with dispersion corrected method (wB97xd) in combination with 3-21G* basis set. Isothermal titration calorimetry (ITC) experiment was carried out in water (unbuffered) on a VP-ITC from Microcal, Inc., at 25 °C. The binding equilibria were studied using a cellular Bis•CB8 solution concentration of 0.1 mM for both Bis and CB8, to which hydrocarbon solution was titrated. Typically, 27 consecutive injections were used with 10 μL each, except for the first one which was set to 5 μL; the heat release related to the first injection was not considered in the subsequent fitting, because some diffusion of titrant from the needle tip is unavoidable during the equilibration time preceding the measurement. All solutions were degassed prior to titration. Curve fitting (Origin 7.0 software) was performed according to a one-set-of-sites model (that is, assuming a 1:1 binding stoichiometry between the added guest and the pre-assembled Bis•CB8 receptor). Note that in the instrumental fitting procedures for the ITC titrations the released heat is plotted against the guest molar ratio rather than the guest concentration, such that the absolute concentration of host (Bis•CB8 receptor) is not required to remain constant in the course of the ITC titration, as opposed to conventional optical and NMR titrations.

2. Packing Coefficient Analysis

Table S1. Guest volume (*V*) and their packing coefficients (*PC*) with the different host systems.

guest	*V*/Å^3 [a]	*PC*/%[b]				
		Bis1•CB8	Bis2•CB8	Bis3•CB8	CB6	CB7
methane	29	25	23	20	20	12
ethane	45	39	36	31	32	19
propane	63	55	50	43	44	26
n-butane	80	70	63	55	56	33
cis-butene	74	65	59	51	52	31
trans-butene	74	65	59	51	52	31
isobutane	79	69	63	54	56	33
isobutene	75	66	60	51	53	31
neopentane	96	84	76	66	68	40
cyclopentane	86	75	75	59	61	36
cyclopentene	81	71	71	55	57	33
cyclohexane	102	89	81	70	72	42
benzene	89	78	71	61	62	37
I_2	71	63	57	49	50	30

[a] Obtained from AM1-optimized structures by using the QSAR module of Hyperchem. [b] *PC* = (guest volume)/(cavity volume); see Table 1 in the main text for the cavity size of the hosts.

3. Fluorescence Binding Titrations

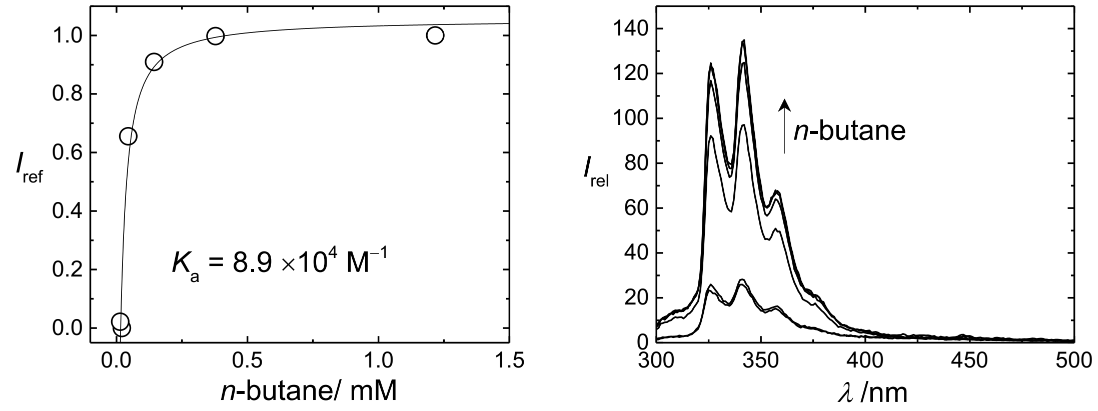

Figure S1. Fluorescence titration of *n*-butane (different pressures) with CB8•Bis2 (10 μM) in aqueous solution.

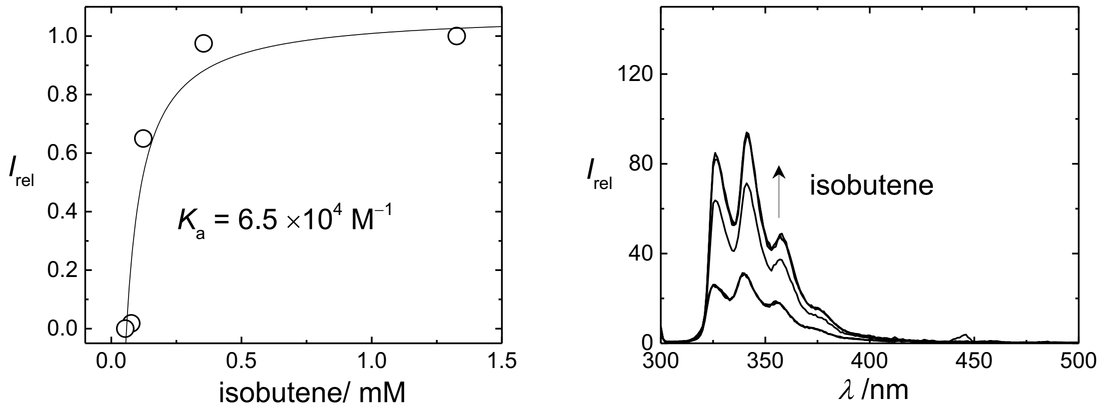

Figure S2. Fluorescence titration of isobutene (different pressures) with CB8•Bis2 (10 μM) in aqueous solution.

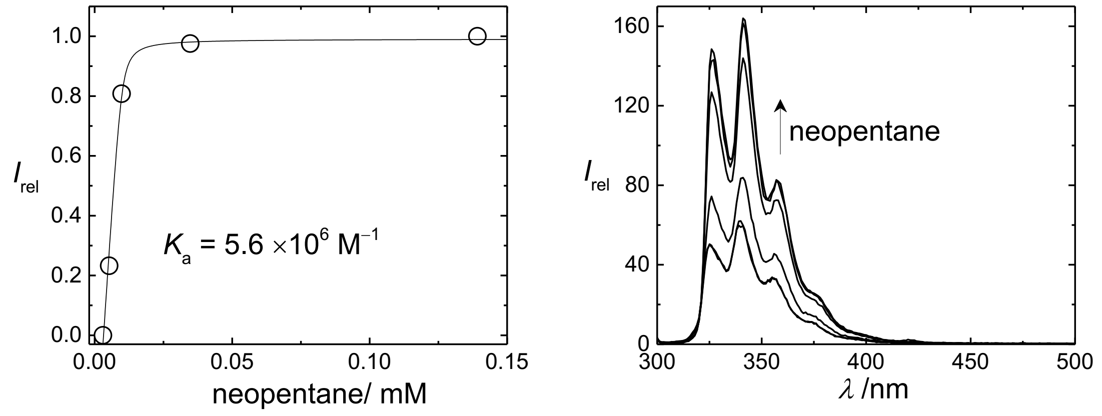

Figure S3. Fluorescence titration of neopentane (different pressures) with CB8•Bis2 (10 μM) in aqueous solution.

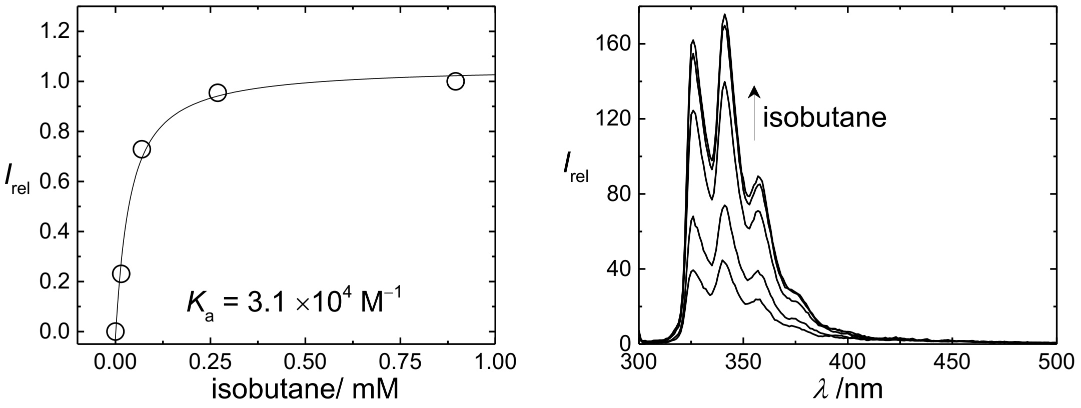

Figure S4. Fluorescence titration of isobutane (different pressures) with CB8•Bis2 (10 μM) in aqueous solution.

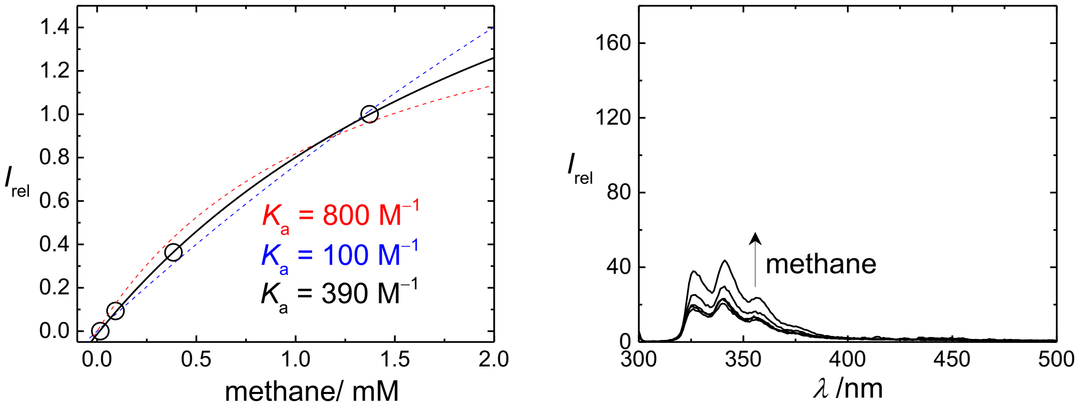

Figure S5. Fluorescence titration of methane (different pressures) with CB8•Bis2 (10 μM) in aqueous solution. Since the plateau region could not be reached in the titration, we also tested significantly lower (100 M^{-1}, blue dashed fitting line) and higher (800 M^{-1}, red dashed line) binding constants to illustrate the robustness of the measurements.

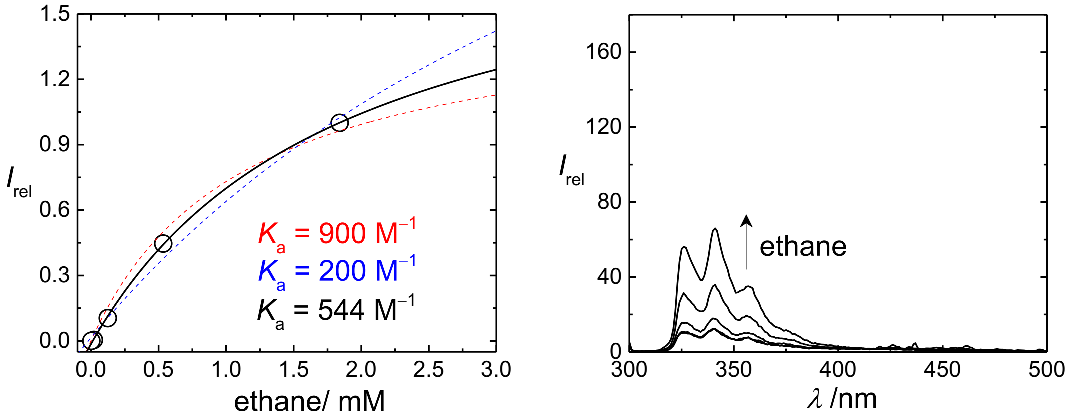

Figure S6. Fluorescence titration of ethane (different pressures) with CB8•Bis2 (10 μM) in aqueous solution. Since the plateau region could not be reached in the titration, we also tested significantly lower (200 M^{-1}, blue dashed fitting line) and higher (900 M^{-1}, red dashed line) binding constants to illustrate the robustness of the measurements.

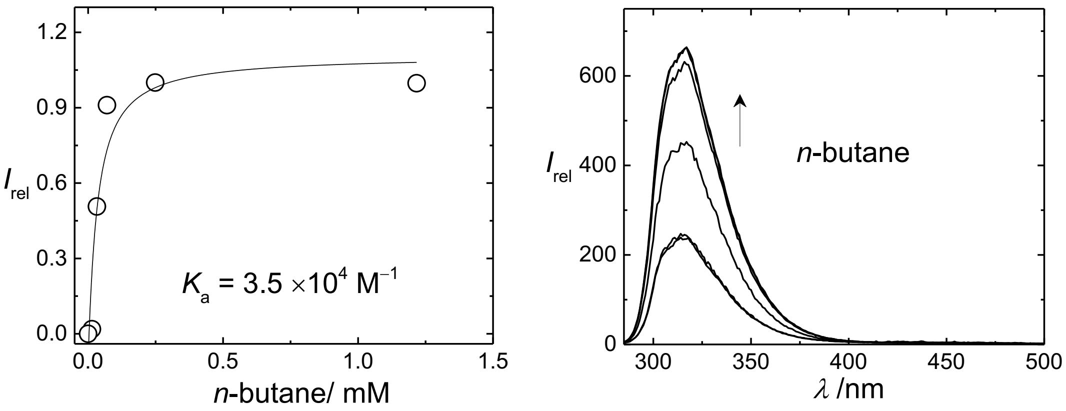

Figure S7. Fluorescence titration of *n*-butane (different pressures) with CB8•Bis3 (10 μM) in aqueous solution.

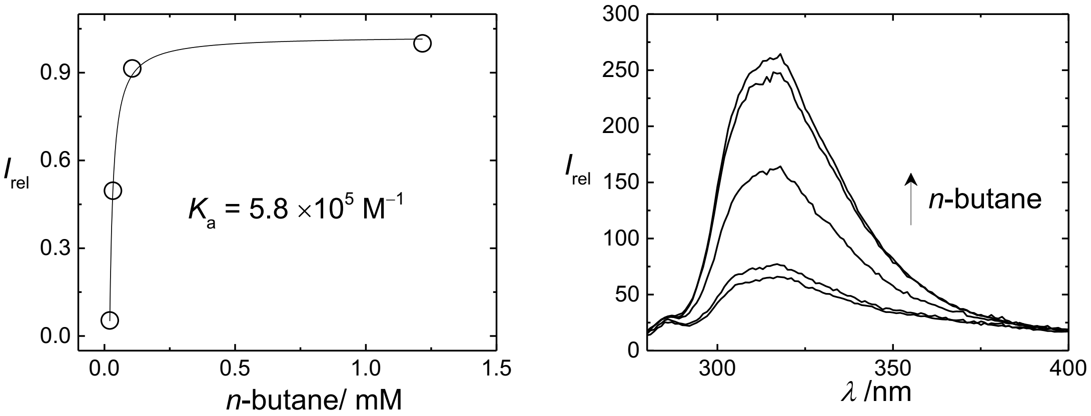

Figure S8. Fluorescence titration of *n*-butane (different pressures) with CB8•Bis1 (10 μM) in aqueous solution.

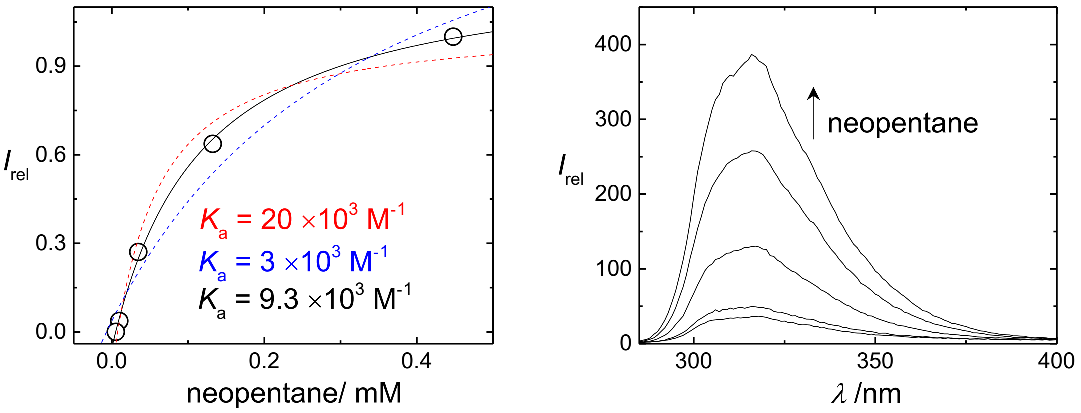

Figure S9. Fluorescence titration of neopentane (different pressures) with CB8•Bis3 (10 μM) in aqueous solution. Since the plateau region could not be reached in the titration, we also tested significantly lower (3 × 10^3 M^{-1}, blue dashed fitting line) and higher (20 × 10^3 M^{-1}, red dashed line) binding constants to illustrate the robustness of the measurements.

Table S2. Relative experimental fluorescence intensities (I_{rel}) obtained upon binding of investigated hydrocarbons to the different Bis•CB8 complexes.

guest	I_{rel}[a]		
	Bis1•CB8	Bis2•CB8	Bis3•CB8
methane	1.5	2.2	7.3
ethane		5.6	
propane		5.0	
n-butane	3.4	5.2	2.8
cis-butene		2.0	
trans-butene		2.4	
isobutane	2.9	4.0	5.0
isobutene	0.6	3.2	0.6
neopentane	4.1	2.7	10.7
cyclopentane		1.5	
cyclopentene		1.1	
cyclohexane		1.4	
benzene		1.4	

[a] $I_{rel} = I_{final}/I_{initial}$.

4. Isothermal Titration Calorimetry Experiments

Table S3. Association constants (K_a) of selected hydrocarbons with Bis•CB8 systems and the associated thermodynamic parameters.[a]

guest	Bis1•CB8			Bis2•CB8			Bis3•CB8		
	K_a/ 10^3 [b]	ΔH[c]	$T\Delta S$[c]	K_a/ 10^3[b]	ΔH[c]	$T\Delta S$[d]	K_a/ 10^3[b]	ΔH[c]	$T\Delta S$[d]
cyclopentene	290	−9.8	−2.4	480 [260][d]	−11.6	−3.8	960 ± 260	−7.9	0.3
1,3-cyclohexadiene	88	−9.9	−3.2	530	−12.4	−4.6	1900 ± 800	−7.5	1.1
benzene	85	−7.6	−0.9	520 [170][d]	−11.7	−3.9	710 ± 230	−8.2	−0.2
cyclopentanol	2.0	−5.4	−0.9	6.7	−7.9	−2.7	5.9	−7.3	−2.2
phenol	3.0	−8.4	−3.7	32 ± 5	−8.7	−2.6	18	−7.7	−1.9

[a] Measured by ITC in water at 25 °C and analyzed for a 1:1 complexation model; 10% errors in K_a and ±0.1 kcal mol^{-1} in ΔH and $T\Delta S$, unless stated differently. [b] Value in M^{-1}. [c] Value in kcal mol^{-1}. [d] Value measured by fluorescence titrations.

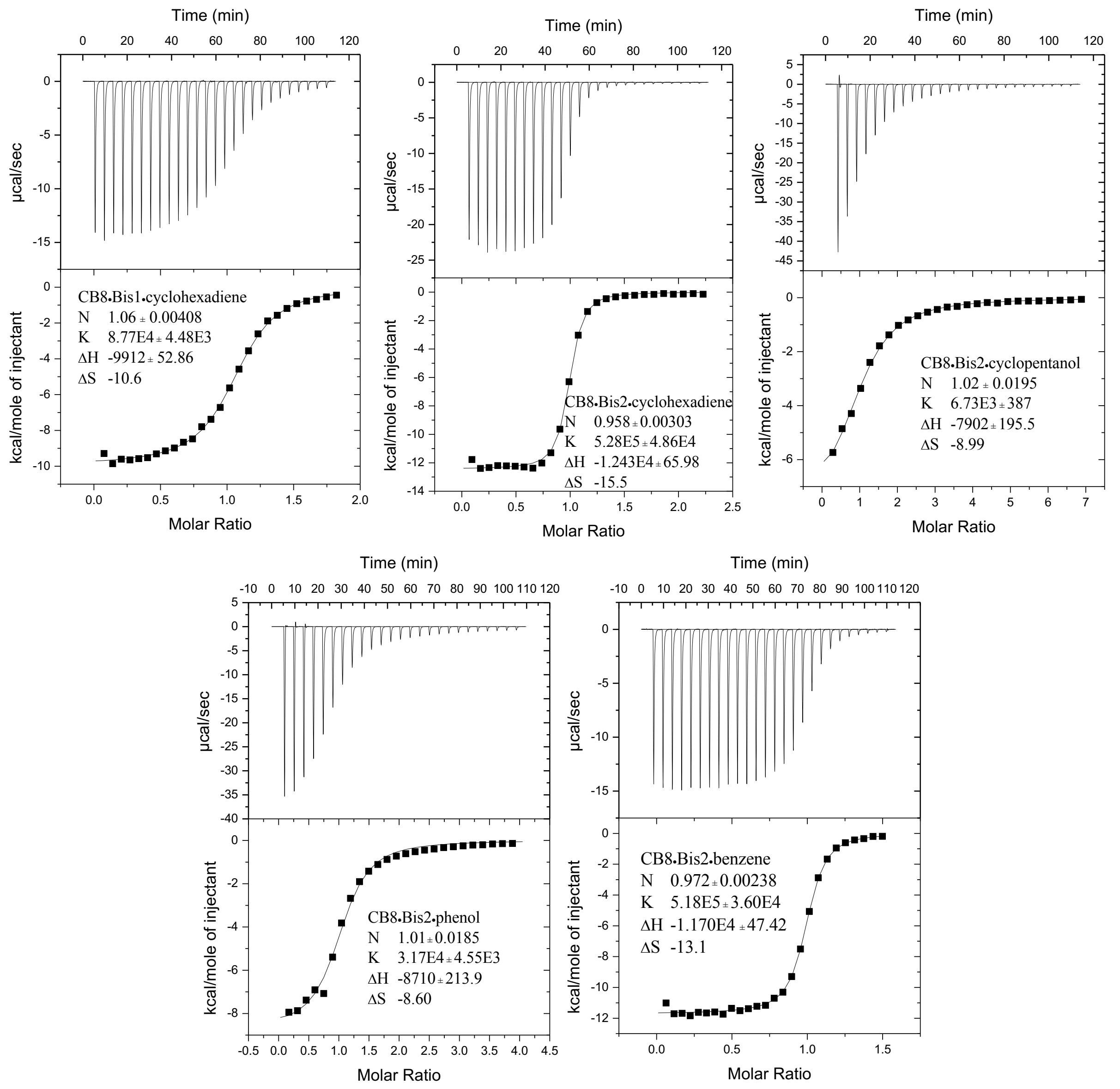

Figure S10. Microcalorimetric titration results in neat water: Raw ITC data for sequential injections of hydrocarbons into Bis•CB8 solution (0.1 mM) and apparent reaction heats obtained from the integration of the calorimetric traces.

Table S4. Guest hydration free energy (ΔG_{hydr}, kcal mol^{-1}), binding free energy (ΔG_a, kcal mol^{-1}), and guest-desolvation corrected binding free energy ($\Delta G_a'$) for the guests studied by ITC.

guest	ΔG_{hydr} [a]	Bis1•CB8		Bis2•CB8		Bis3•CB8	
		ΔG_a [b]	$\Delta G_a'$ [c]	ΔG_a [b]	$\Delta G_a'$ [c]	ΔG_a [b]	$\Delta G_a'$ [c]
cyclopentene	0.55	−7.45	−6.90	−7.75	−7.20	−8.2±0.2	−7.6
1,3-cyclohexadiene	−0.39	−6.74	−7.13	−7.81	−8.20	−8.6±0.2	−9.0
benzene	−0.89	−6.72	−7.61	−7.80	−8.69	−8.0±0.2	−8.9
cyclopentanol	−5.49[d]	−4.50	−9.99	−5.22	−10.71	−5.14	−10.63
phenol	−6.66[d]	−4.74	−11.40	−6.15	−12.81	−5.80	−12.46

[a] Calculated from tabulated aqueous solubilities (S) and vapor pressures (p_{vap}) at 298 K according to $\Delta G_{hydr} = -RT\ln(S*p^0/p_{vap})$, with p_{vap} in kPa and p^0 = 101.325 kPa. [b] Calculated from the K_a values obtained by ITC in Table S3; error ±0.10 kcal mol^{-1}, unless stated differently. [c] $\Delta G_a' = \Delta G_a + \Delta G_{hydr}$. [d] From ref. 2.

5. Diiodine Binding to the Bis•CB8

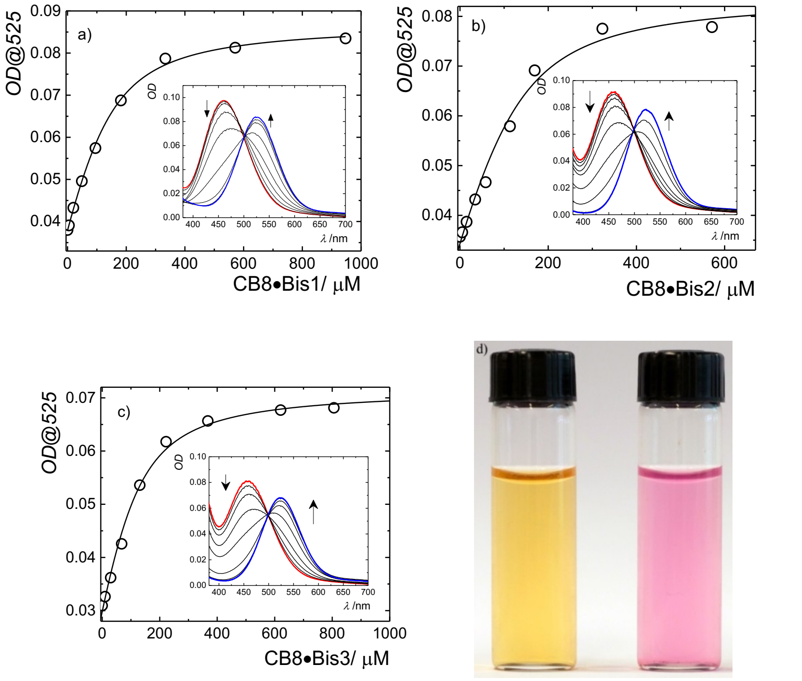

Figure S11. UV-visible titrations of iodine (100-130 μM) with a) Bis1•CB8, b) Bis2•CB8, and c) Bis3•CB8 in aqueous solution. d) Color change of I_2 from yellow-brown (known in water) to violet (known in nonpolar solvents) upon complexation with Bis•CB8 complexes. $K_{Bis1•CB8}$ = (1.7 ± 0.2) × 10^4 M^{-1}; $K_{Bis2•CB8}$ = (2.1 ± 0.8) × 10^4 M^{-1}; $K_{Bis3•CB8}$ = (1.9 ± 0.4) × 10^4 M^{-1}.

6. NMR Studies

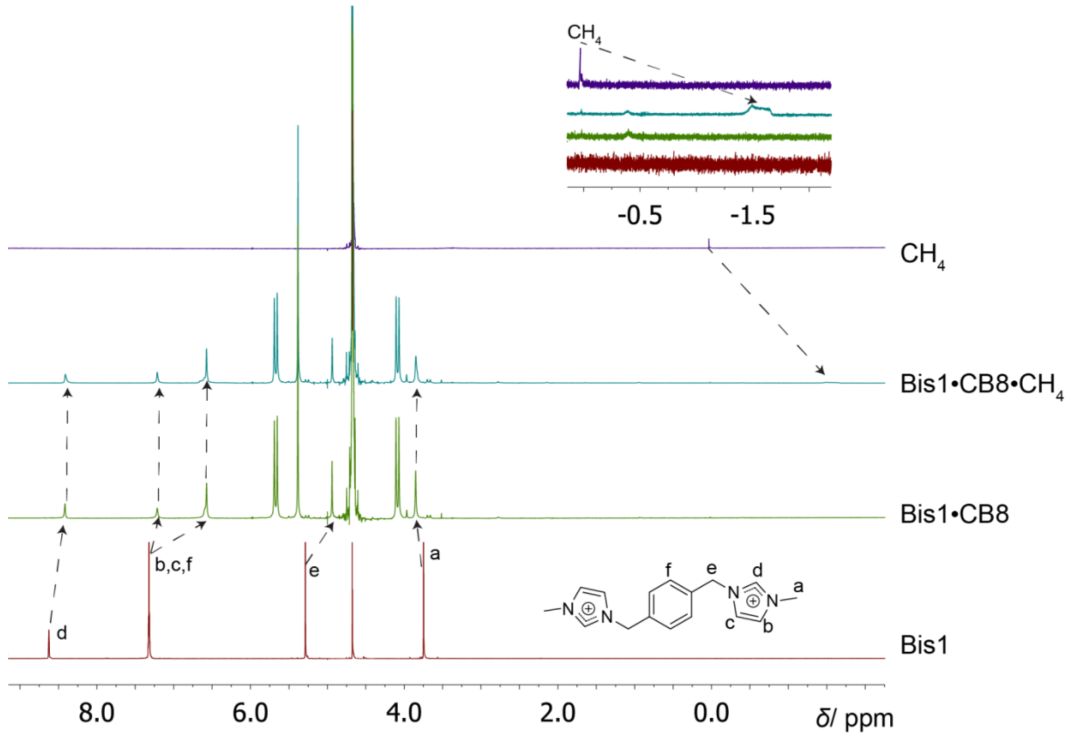

Figure S12. ^{1}H NMR spectra of bisimidazolium Bis1, Bis1•CB8 (6.25 mM), Bis1•CB8•CH_4, and CH_4 free.

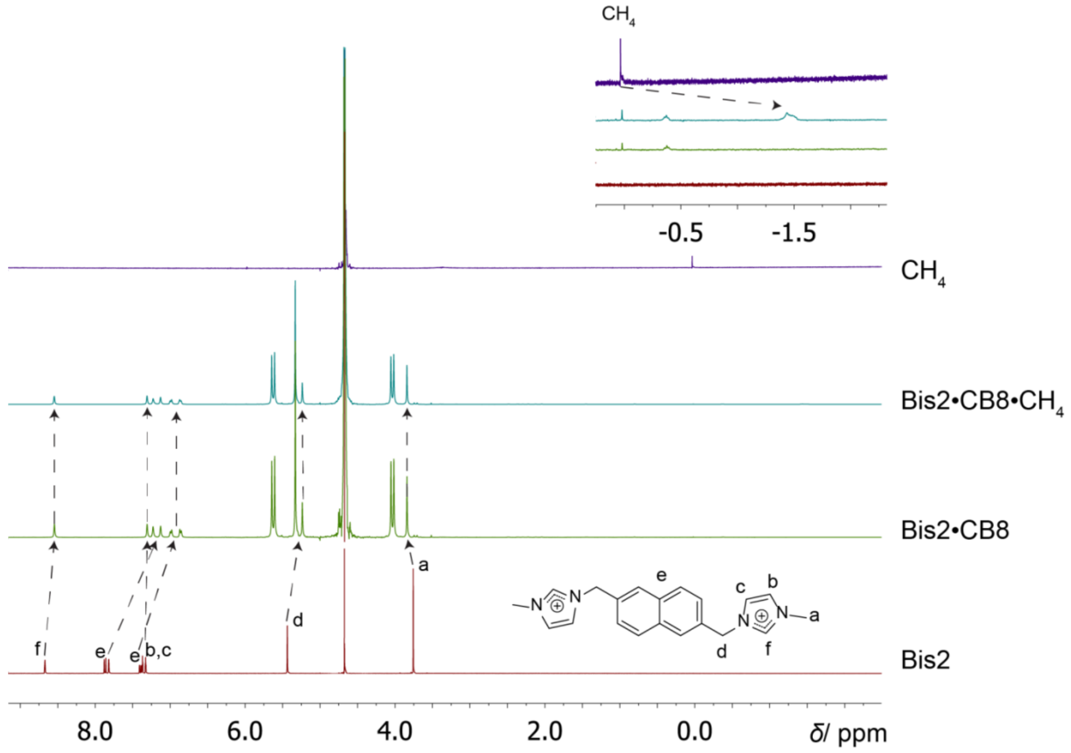

Figure S13. ^{1}H NMR spectra of bisimidazolium Bis2, Bis2•CB8 (6.25 mM), Bis2•CB8•CH_4, and CH_4 free.

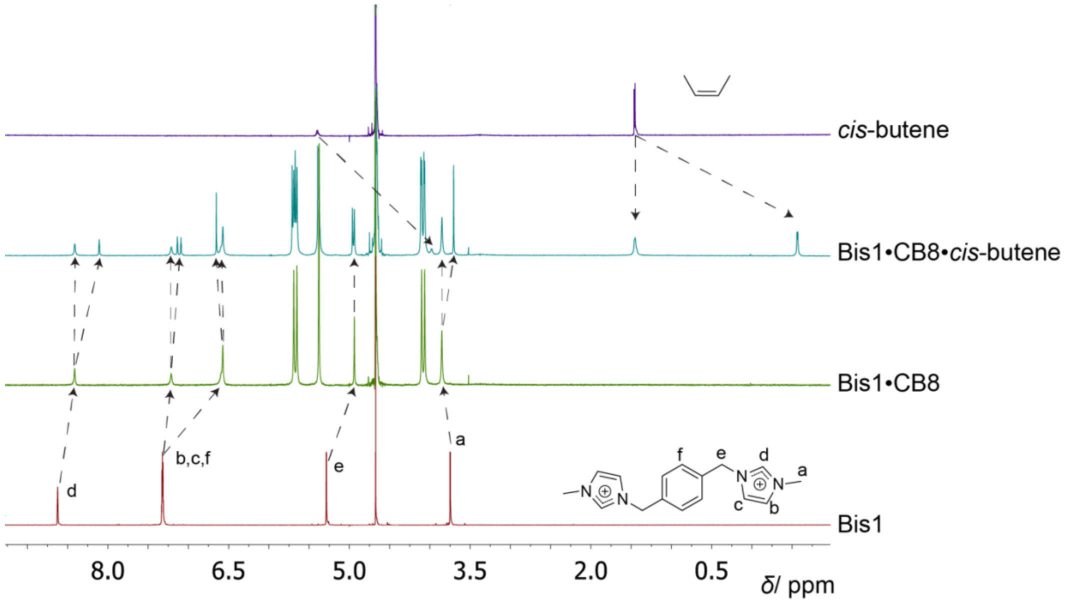

Figure S14. ^{1}H NMR spectra of bisimidazolium Bis1, Bis1•CB8 (1.25 mM), Bis1•CB8•*cis*-butene, and *cis*-butene free.

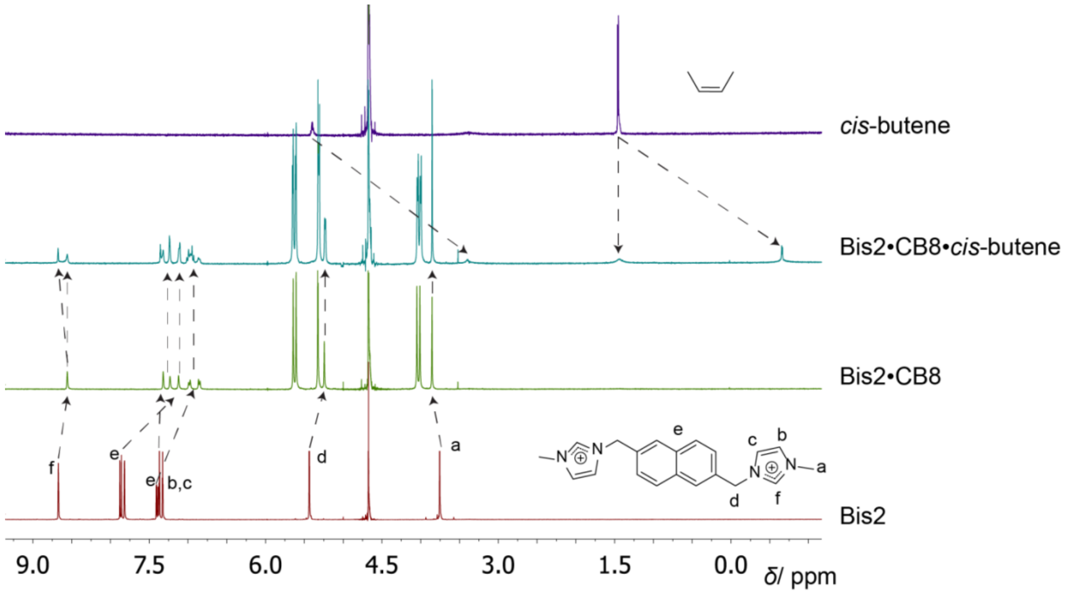

Figure S15. ^{1}H NMR spectra of bisimidazolium Bis2, Bis2•CB8 (1.25 mM), Bis2•CB8•*cis*-butene, and *cis*-butene free.

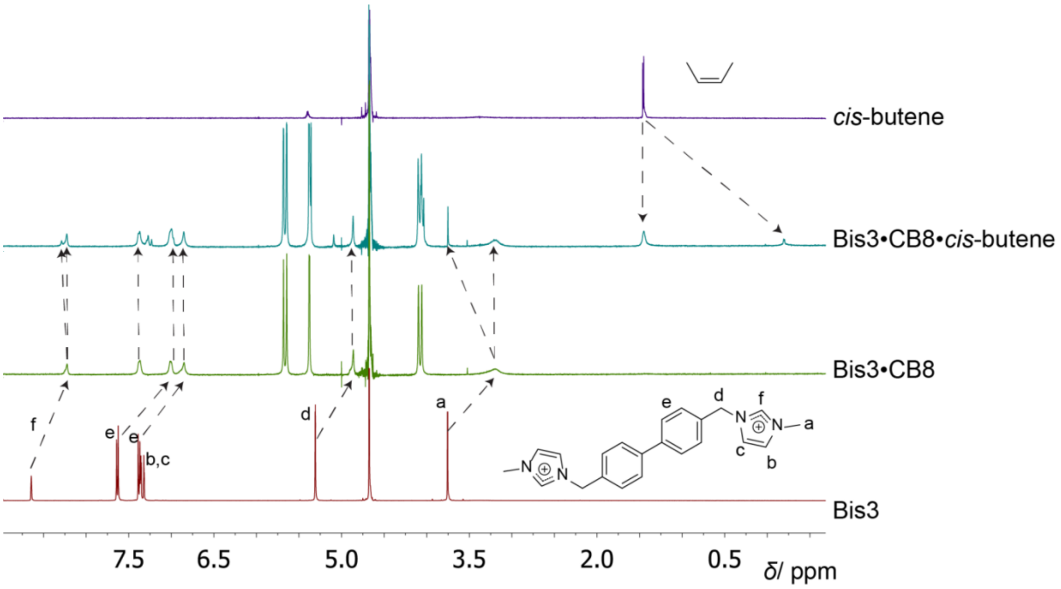

Figure S16. ^{1}H NMR spectra of bisimidazolium Bis3, Bis3•CB8 (1.25 mM), Bis3•CB8•*cis*-butene, and *cis*-butene free.

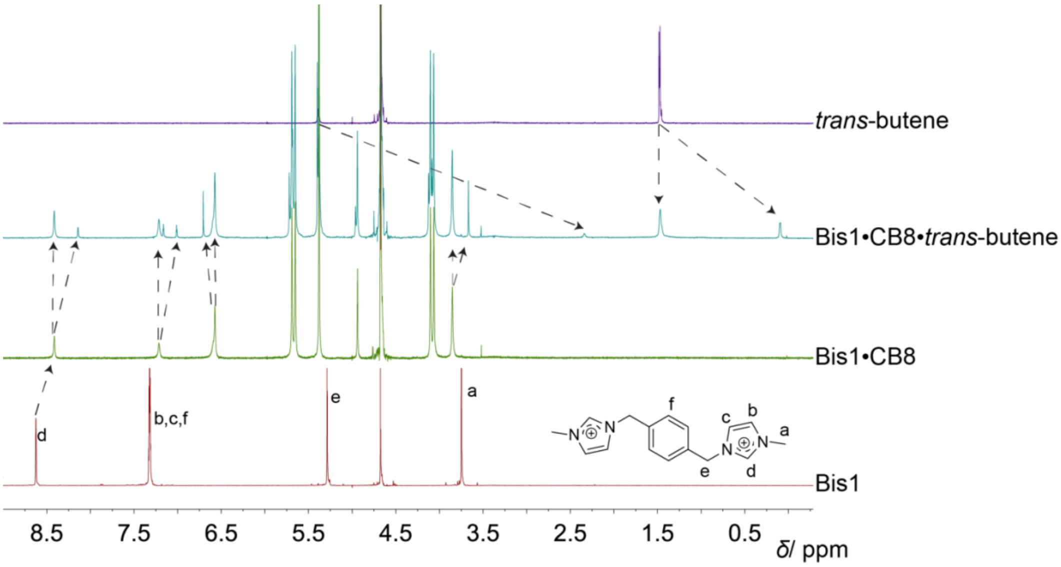

Figure S17. ^{1}H NMR spectra of bisimidazolium Bis1, Bis1•CB8 (1.25 mM), Bis1•CB8•*trans*-butene, and *trans*-butene free.

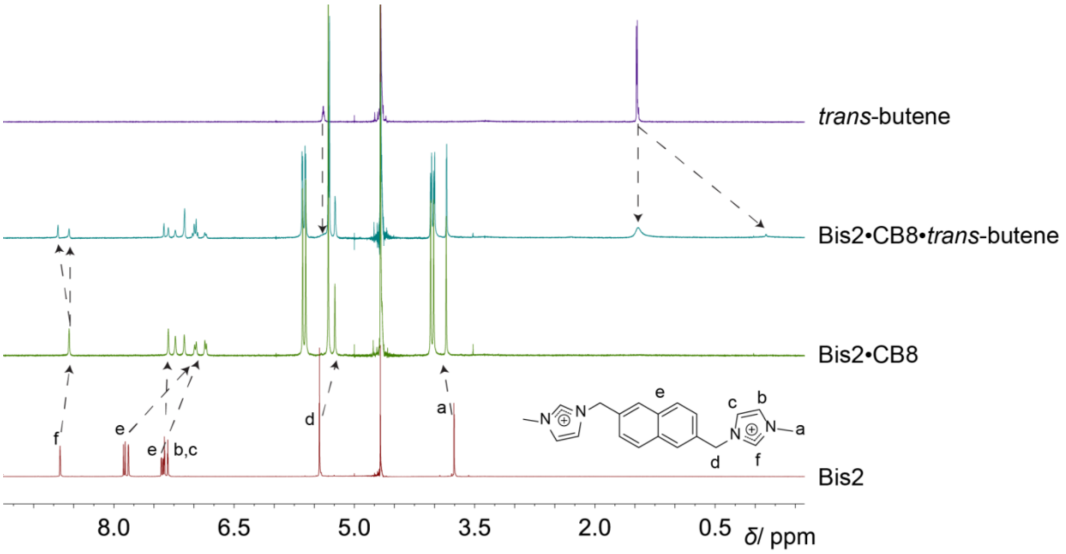

Figure S18. ^{1}H NMR spectra of bisimidazolium Bis2, Bis2•CB8 (1.25 mM), Bis2•CB8•*trans*-butene, and *trans*-butene free.

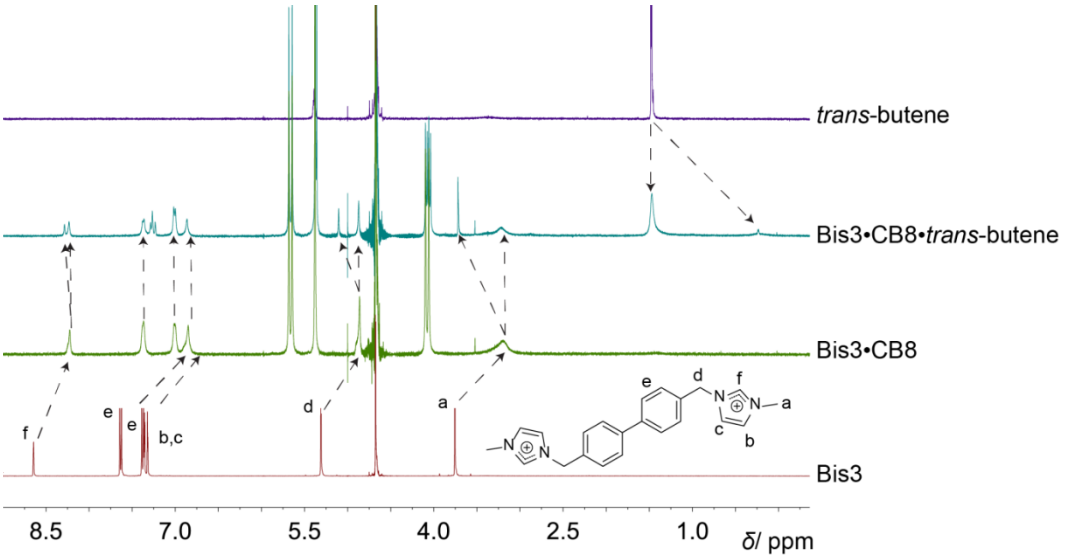

Figure S19. ^{1}H NMR spectra of bisimidazolium Bis3, Bis3•CB8 (1.25 mM), Bis3•CB8•*trans*-butene, and *trans*-butene free.

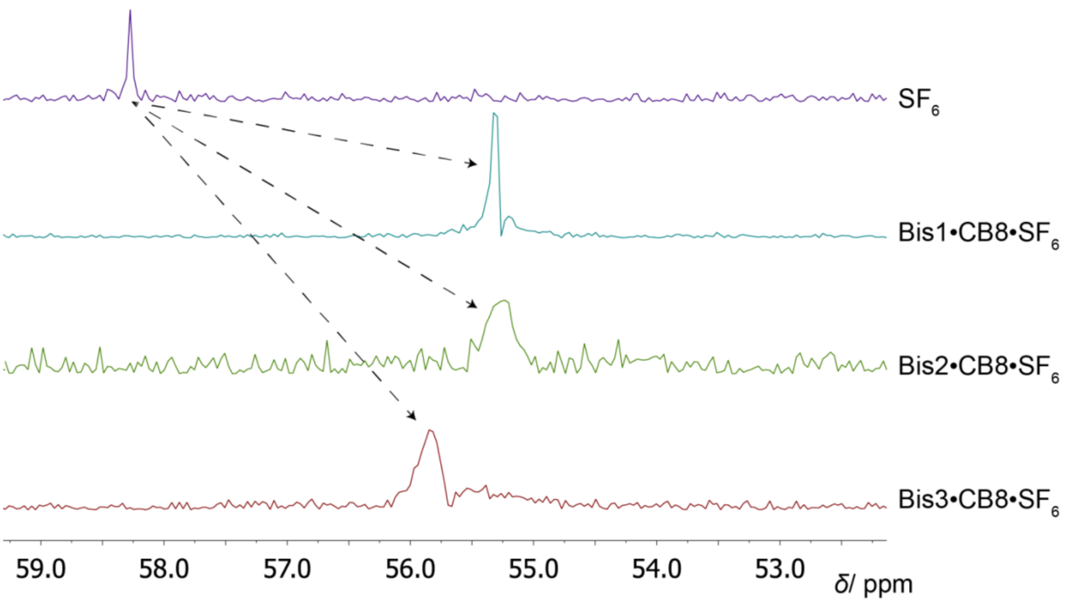

Figure S20. ^{19}F NMR spectra of (1.25 mM) Bis1•CB8•SF_6, Bis2•CB8•SF_6, Bis3•CB8•SF_6, and SF_6 free. The signal broadening is related to the guest exchange kinetics.

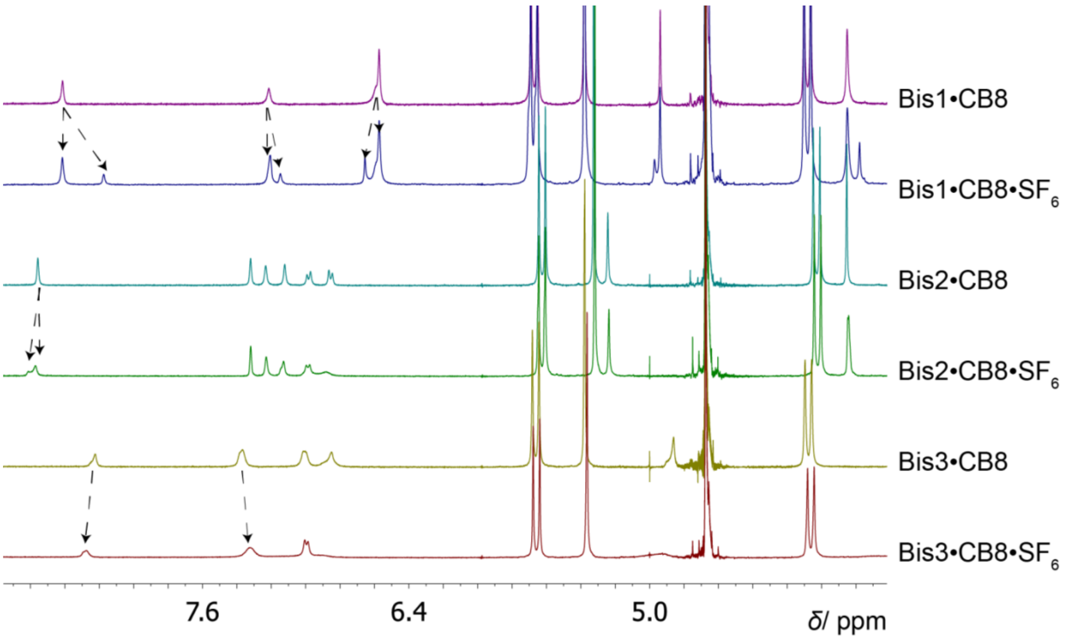

Figure S21. ^{1}H NMR spectra of (1.25 mM) Bis1•CB8, Bis2•CB8, Bis3•CB8, Bis1•CB8•SF_6, Bis2•CB8•SF_6, and Bis3•CB8•SF_6.

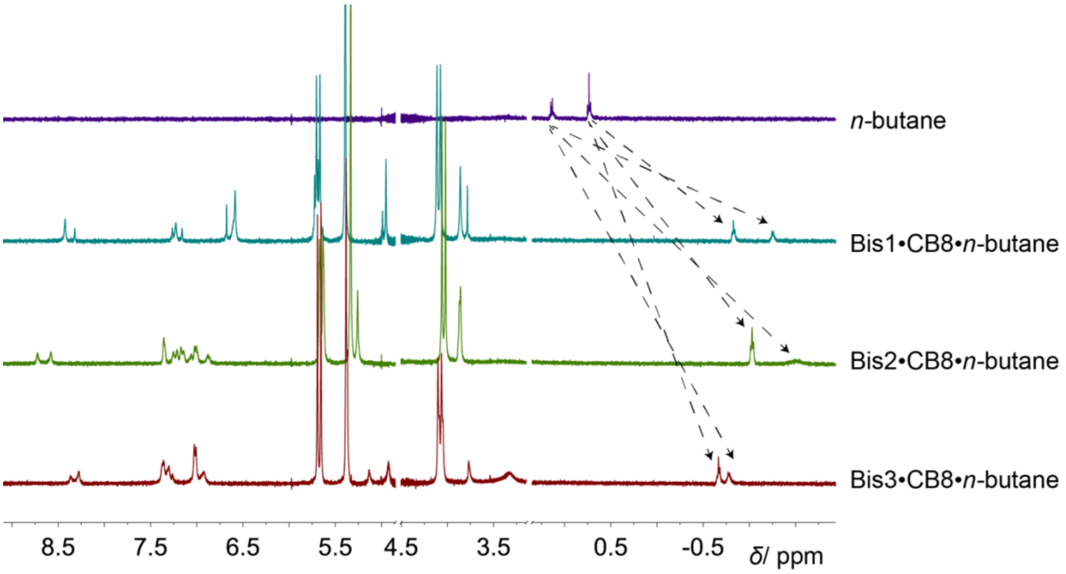

Figure S22. ^{1}H NMR spectra of (1.25 mM) *n*-butane, Bis1•CB8•*n*-butane, Bis2•CB8•*n*-butane, and Bis3•CB8•*n*-butane.

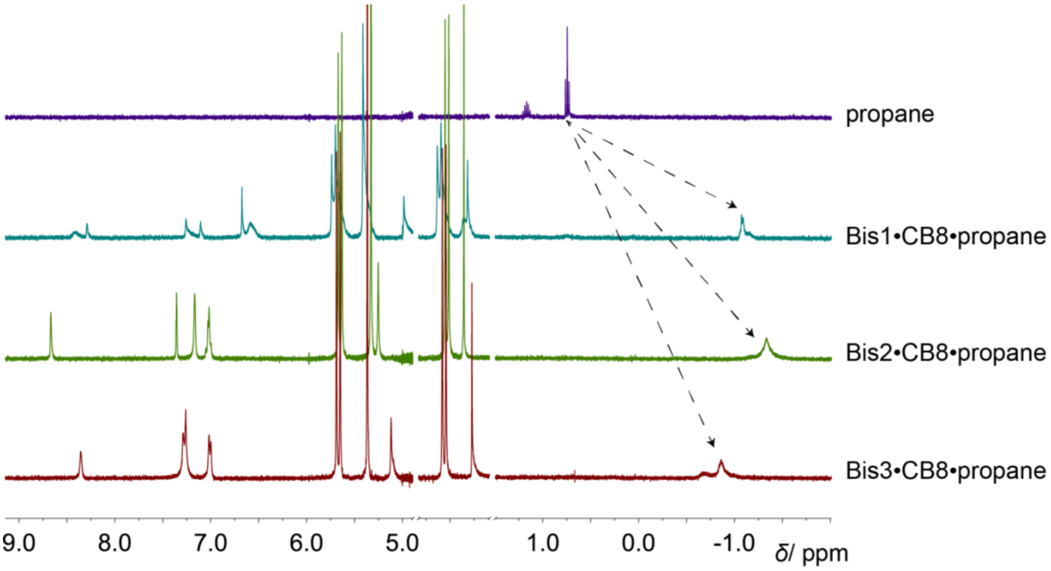

Figure S23. ^{1}H NMR spectra of (1.25 mM) propane, Bis1•CB8•propane, Bis2•CB8•propane, and Bis3•CB8•propane.

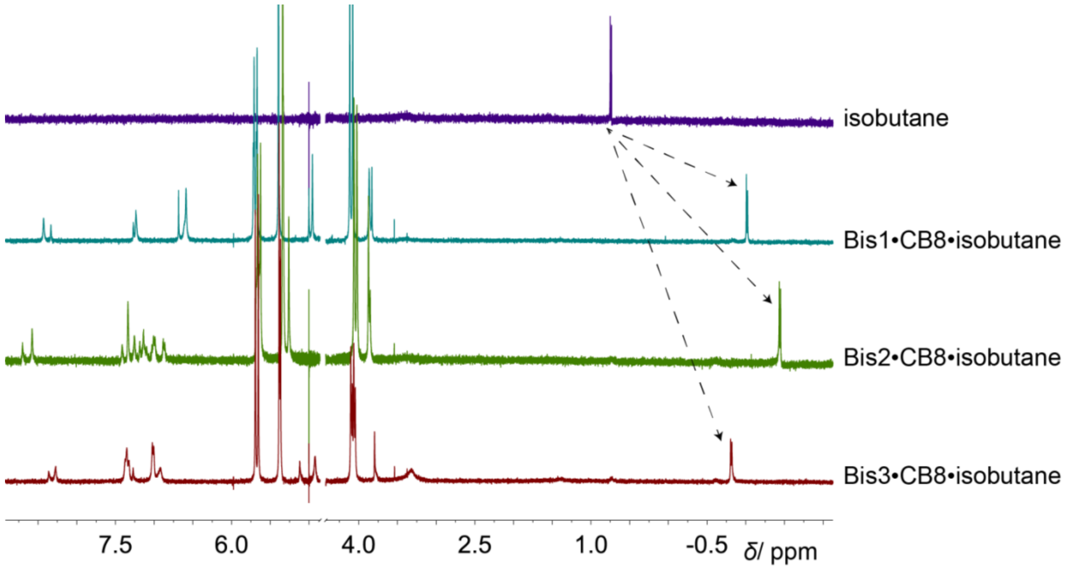

Figure S24. ^{1}H NMR spectra of (1.25 mM) isobutane, Bis1•CB8•isobutane, Bis2•CB8•isobutane, and Bis3•CB8•isobutane.

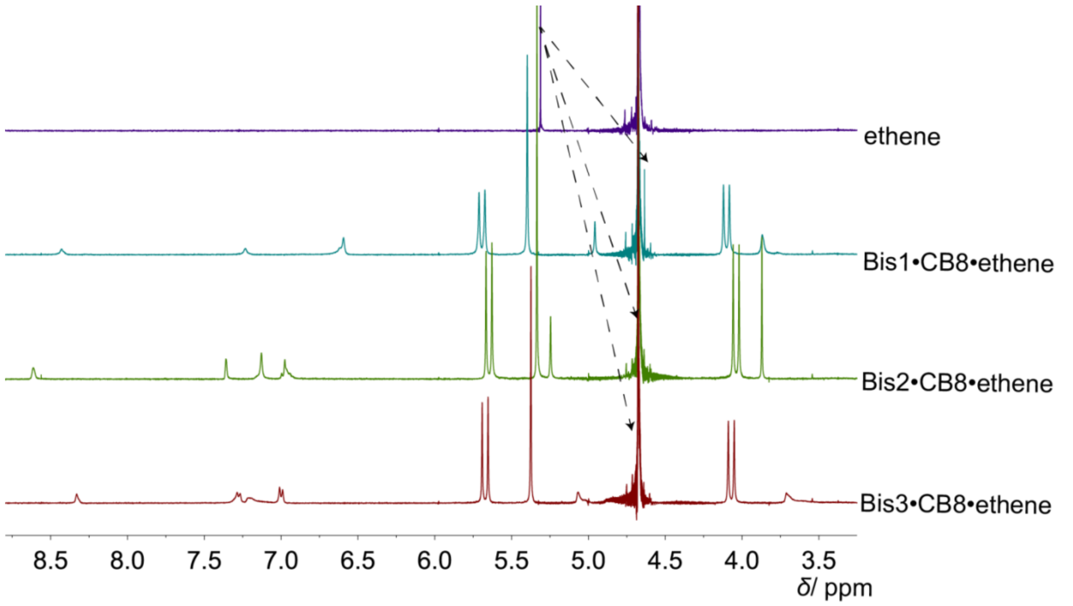

Figure S25. ^{1}H NMR spectra of (1.25 mM) ethene, Bis1•CB8•ethene, Bis2•CB8•ethene, and Bis3•CB8•ethene.

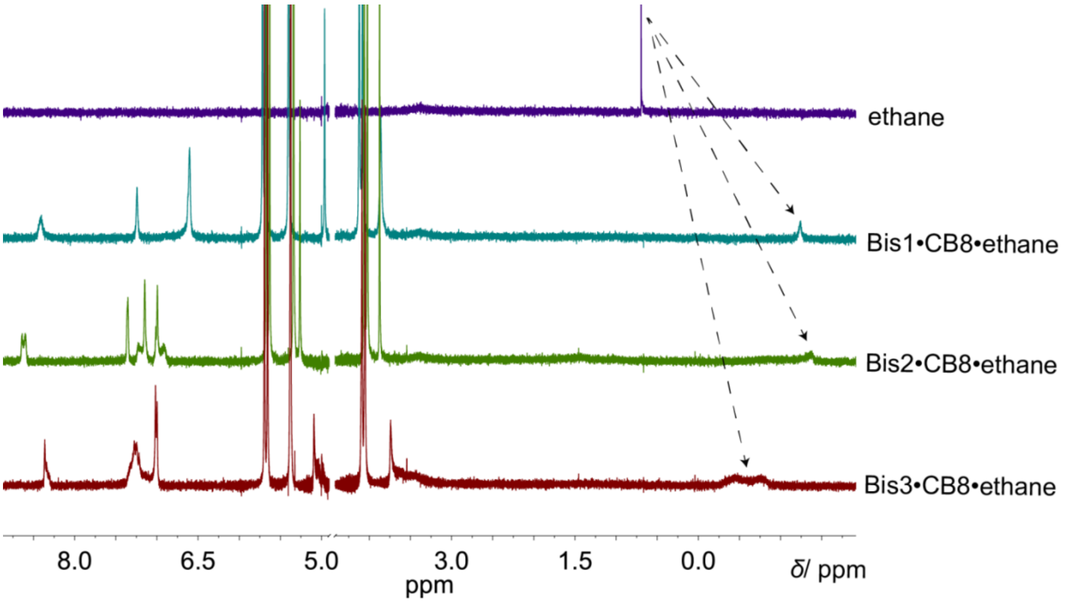

Figure S26. ^{1}H NMR spectra of (1.25 mM) ethane, Bis1•CB8•ethane, Bis2•CB8•ethane, and Bis3•CB8•ethane.

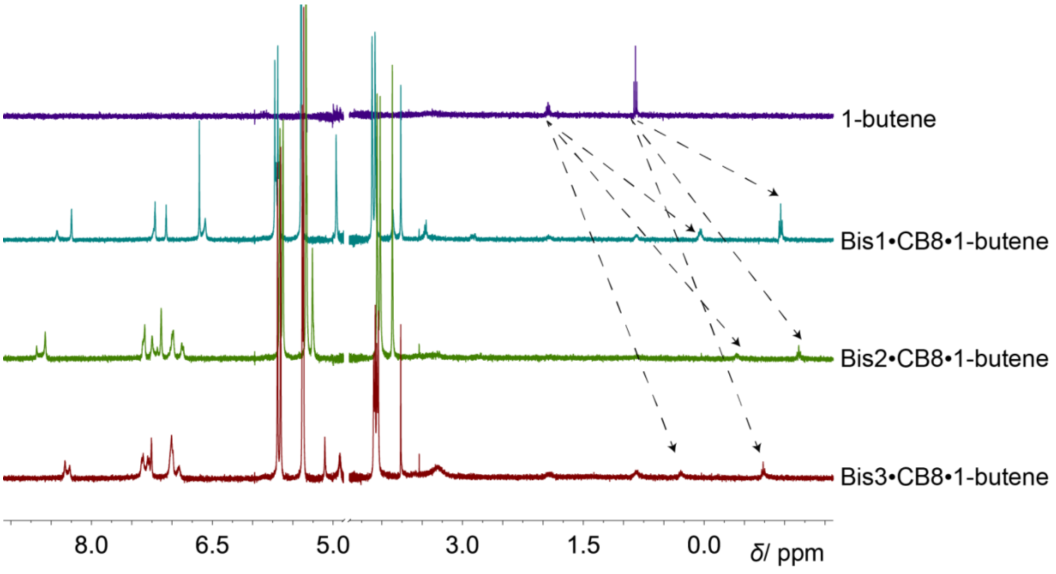

Figure S27. ^{1}H NMR spectra of (1.25 mM) 1-butene, Bis1•CB8•1-butene, Bis2•CB8•1-butene, and Bis3•CB8•1-butene.

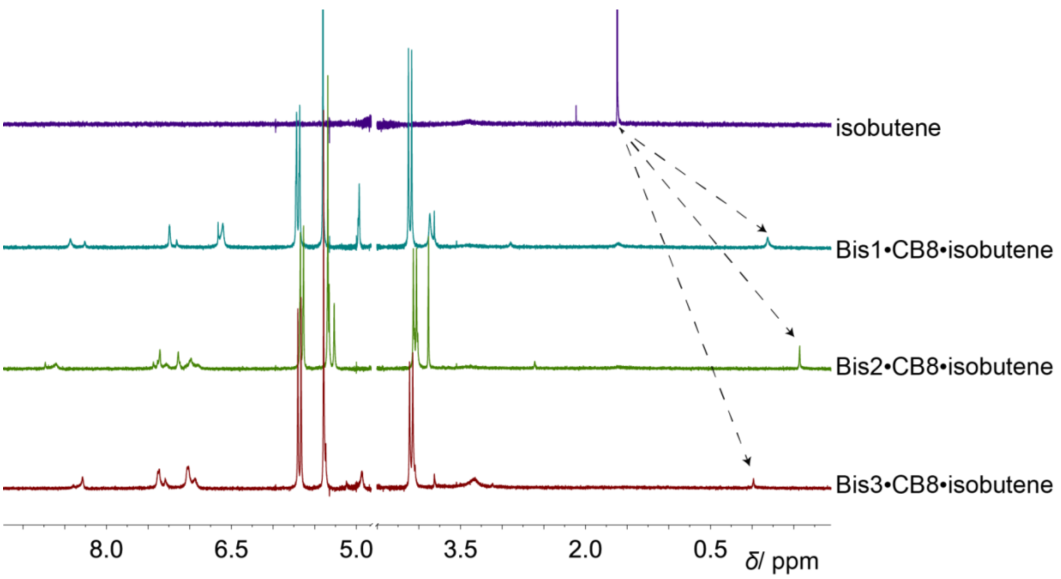

Figure S28. ^{1}H NMR spectra of (1.25 mM) isobutene, Bis1•CB8•isobutene, Bis2•CB8•isobutene, and Bis3•CB8•isobutene.

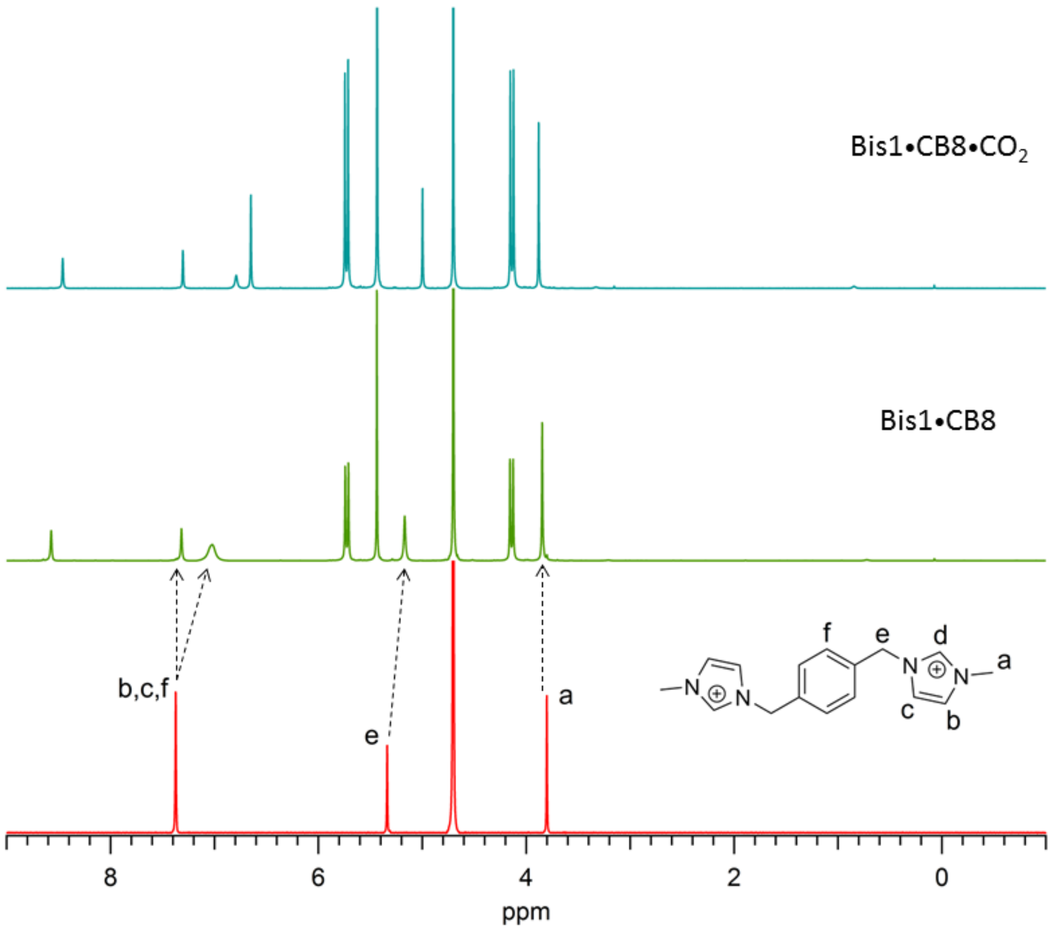

Figure S29. ^{1}H NMR spectra of bisimidazolium Bis1, Bis1•CB8 and Bis1•CB8•CO_2.

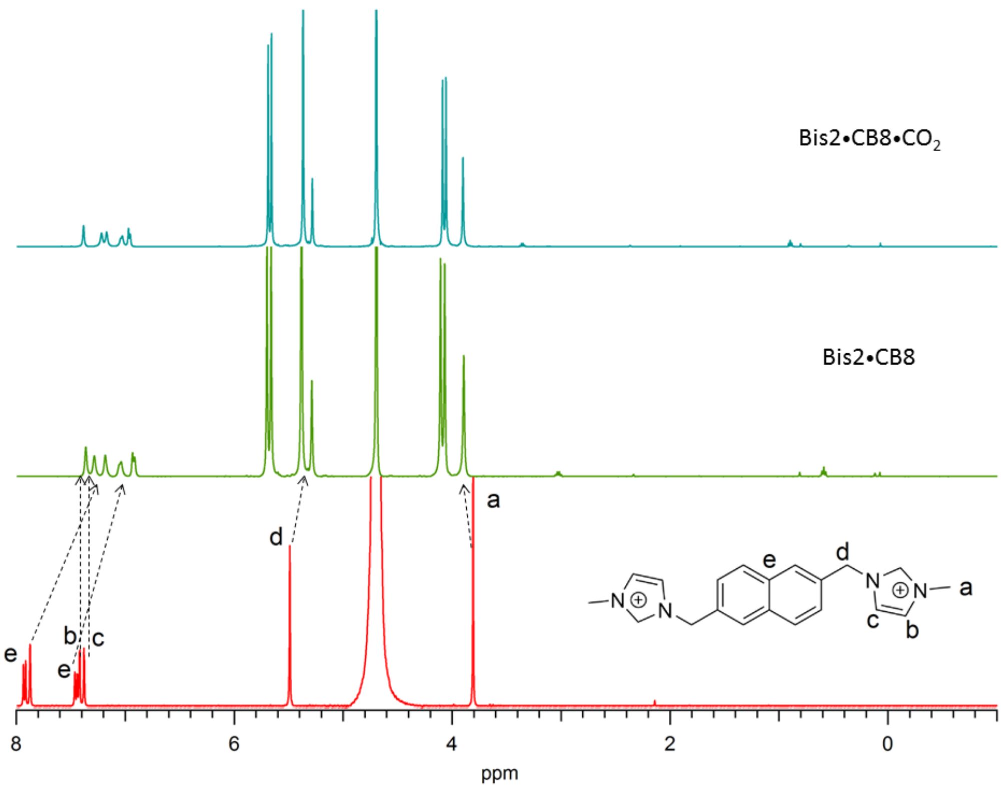

Figure S30. ^{1}H NMR spectra of bisimidazolium Bis2, Bis2•CB8 and Bis2•$CB8•CO_2$.

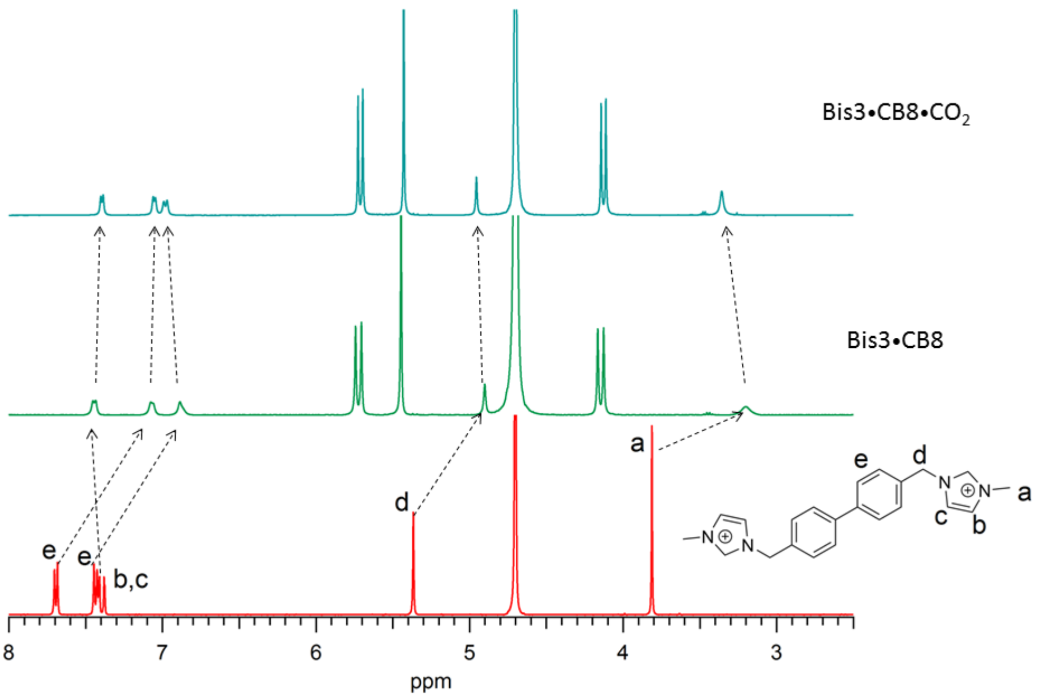

Figure S31. ^{1}H NMR spectra of bisimidazolium Bis3, Bis3•CB8 and Bis3•$CB8•CO_2$.

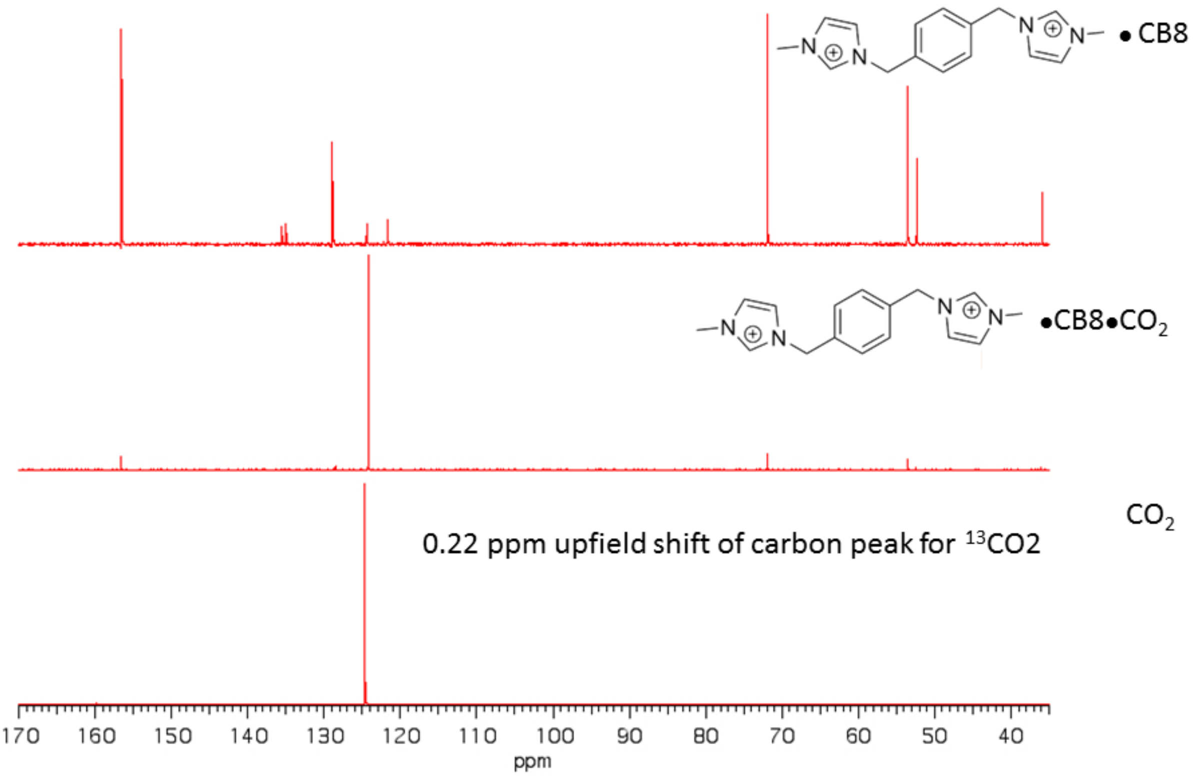

Figure S32. ^{13}C NMR spectra of Bis1•CB8, Bis1•CB8•$^{13}CO_2$ and $^{13}CO_2$.

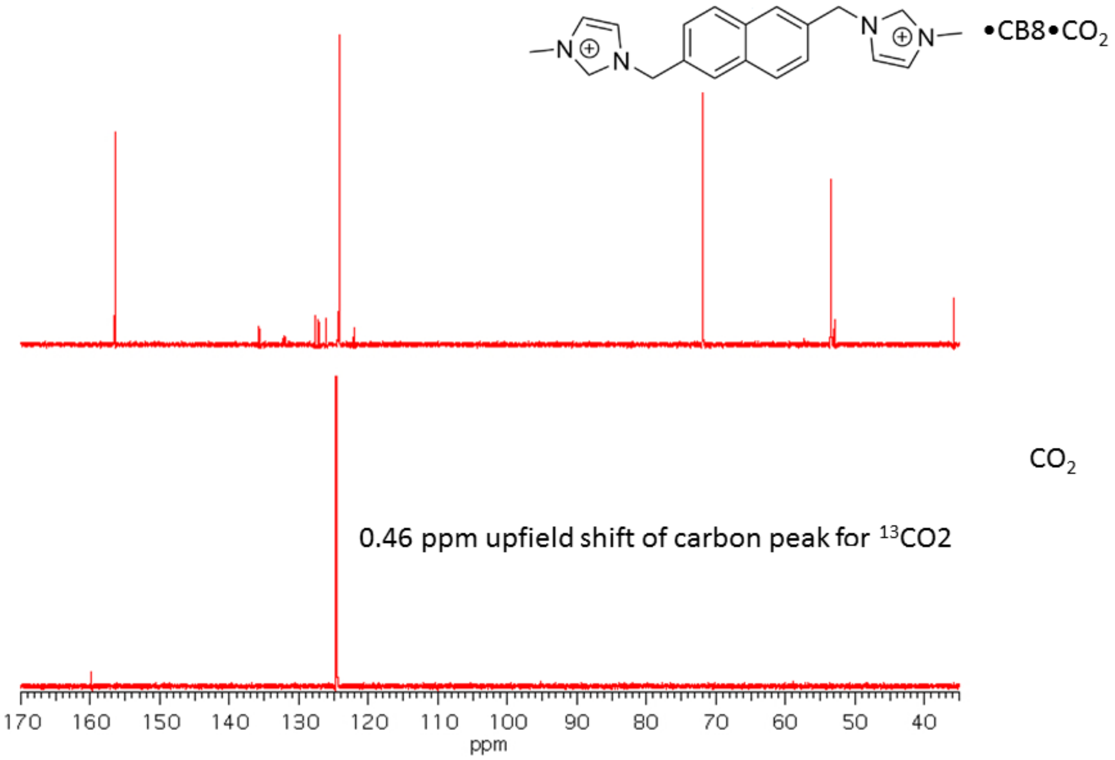

Figure S33. ^{13}C NMR spectra of Bis2•CB8, Bis2•CB8•$^{13}CO_2$ and $^{13}CO_2$.

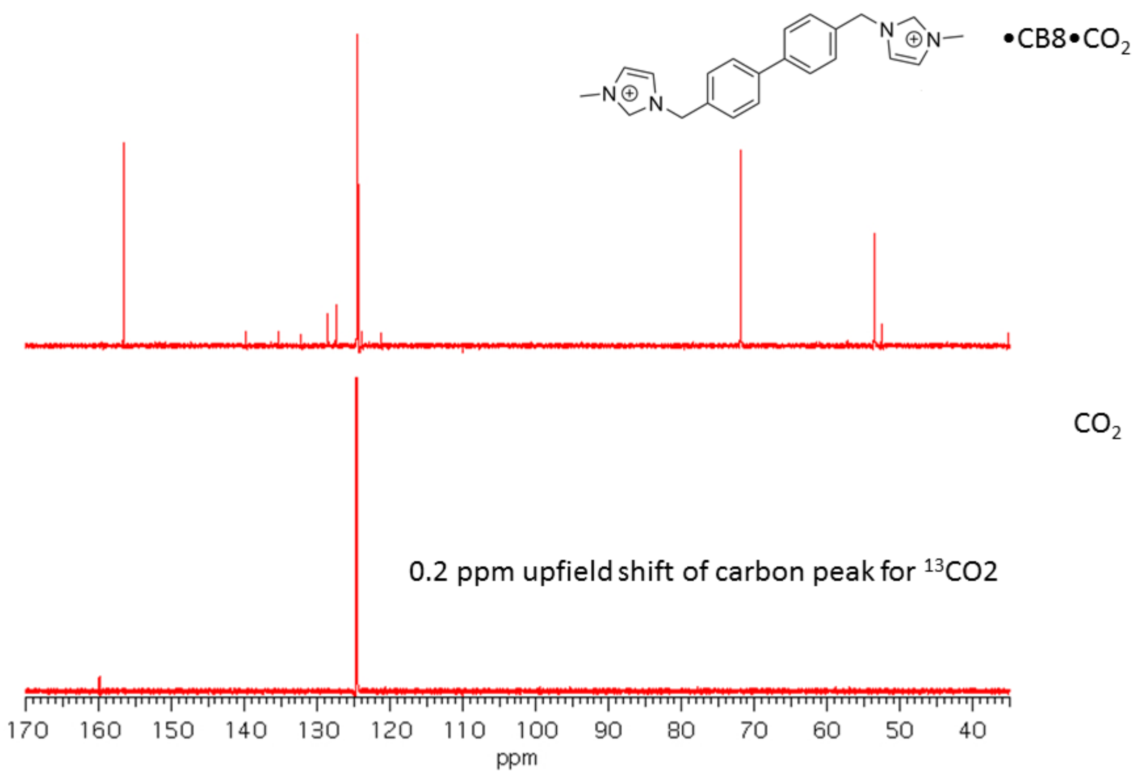

Figure S34. ^{13}C NMR spectra of Bis3•CB8, Bis3•CB8•$^{13}CO_2$ and $^{13}CO_2$.

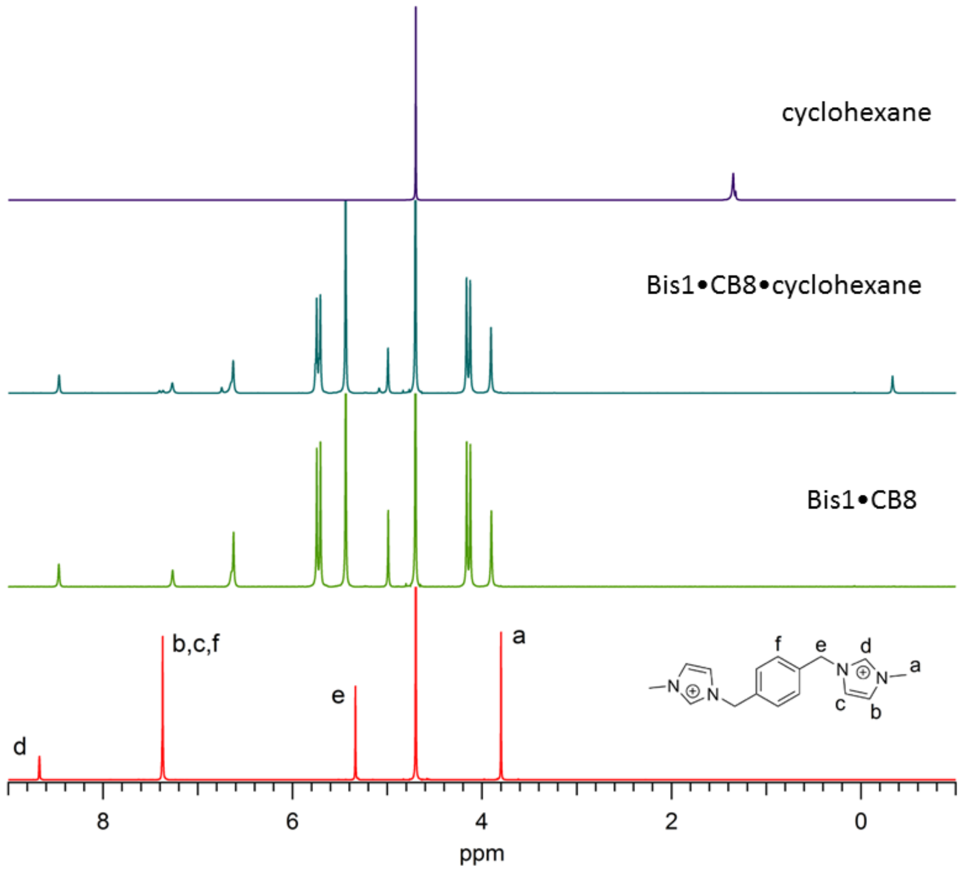

Figure S35. ^1H NMR spectra of bisimidazolium Bis1, Bis1•CB8 (1.25 mM), Bis1•CB8•cyclohexane, and cyclohexane free.

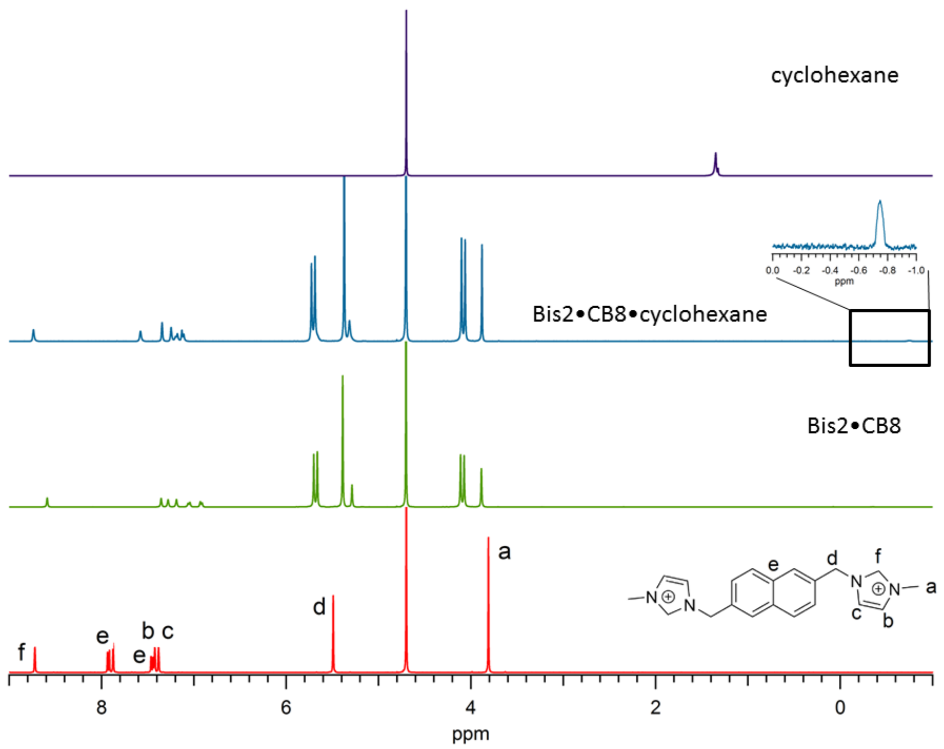

Figure S36. ^{1}H NMR spectra of bisimidazolium Bis2, Bis2•CB8 (1.25 mM), Bis2•CB8•cyclohexane, and cyclohexane free.

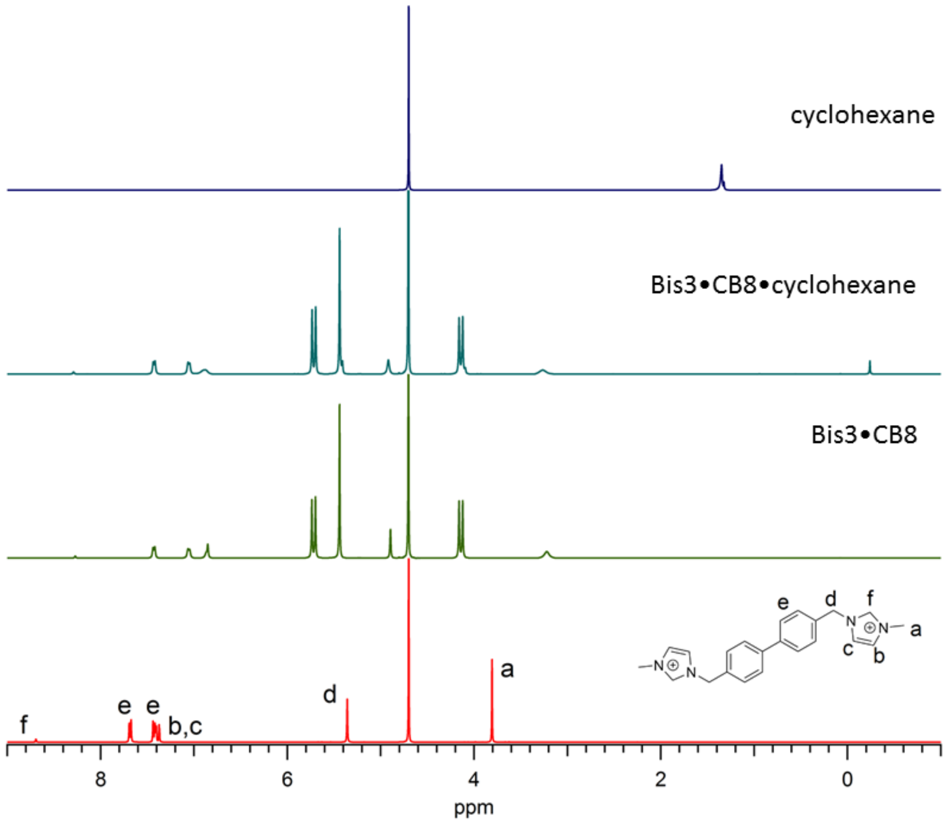

Figure S37. ^{1}H NMR spectra of bisimidazolium Bis3, Bis3•CB8 (1.25 mM), Bis3•CB8•cyclohexane, and cyclohexane free.

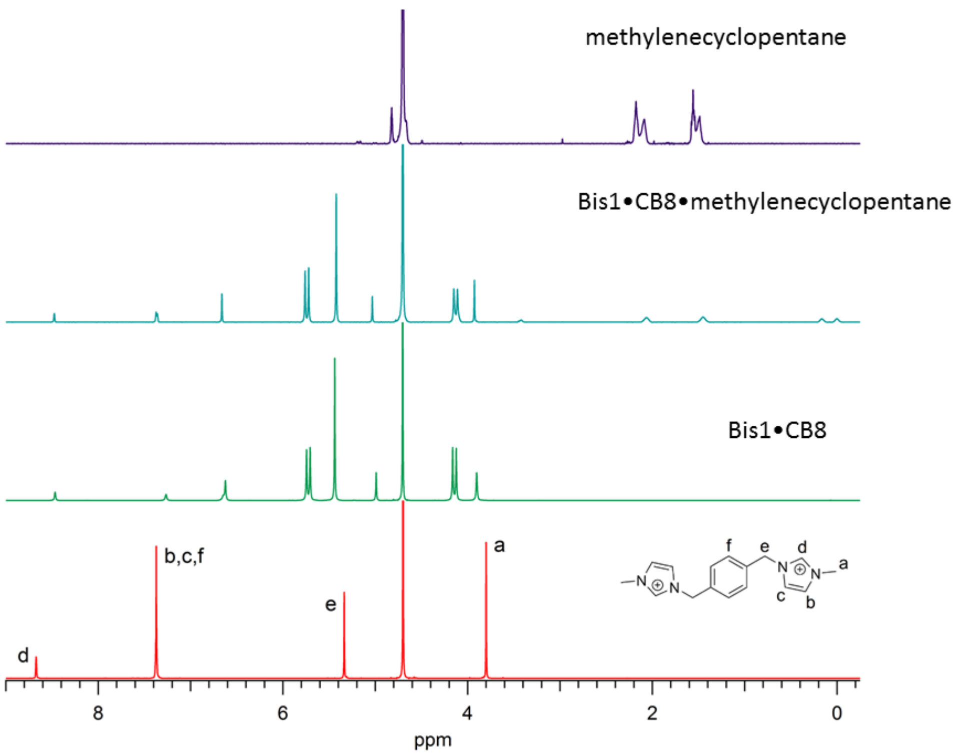

Figure S38. ^{1}H NMR spectra of bisimidazolium Bis1, Bis1•CB8 (1.25 mM), Bis3•CB8•methylenecyclopentane, and methylenecyclopentane free.

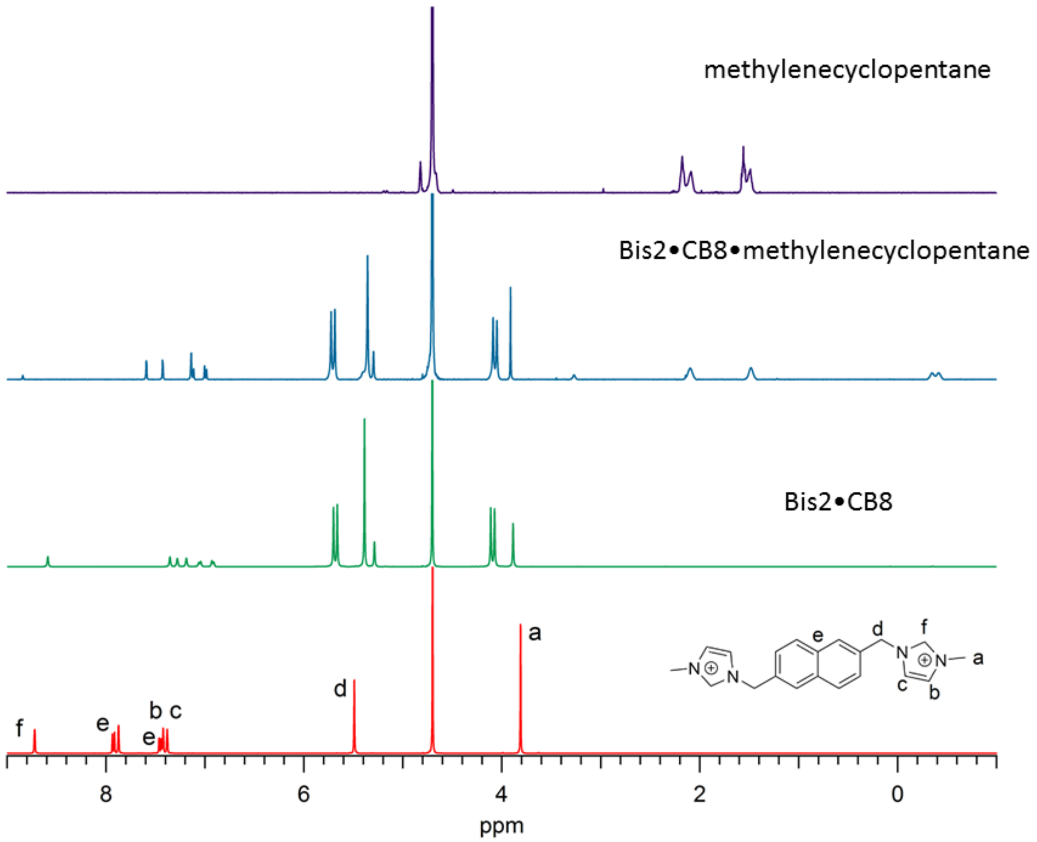

Figure S39. ^{1}H NMR spectra of bisimidazolium Bis2, Bis2•CB8 (1.25 mM), Bis2•CB8•methylenecyclopentane, and methylenecyclopentane free.

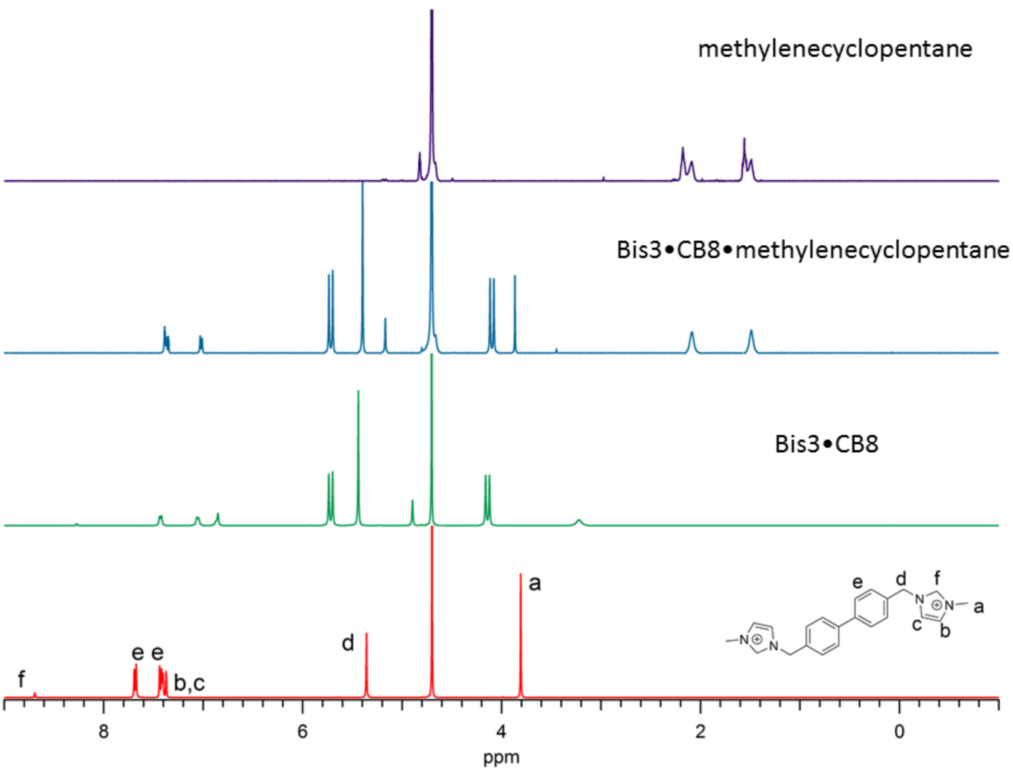

Figure S40. ^{1}H NMR spectra of bisimidazolium Bis3, Bis3•CB8 (1.25 mM), Bis3•CB8•methylenecyclopentane, and methylenecyclopentane free.

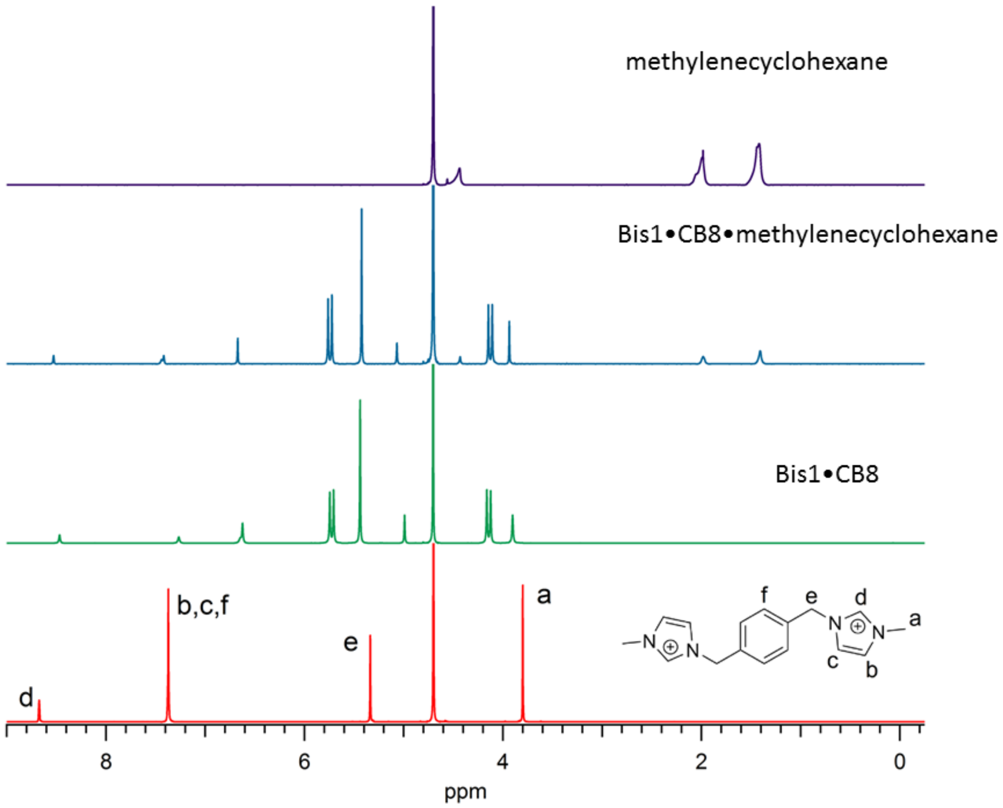

Figure S41. ^{1}H NMR spectra of bisimidazolium Bis1, Bis1•CB8 (1.25 mM), Bis1•CB8•methylenecyclohexane, and methylenecyclohexane free.

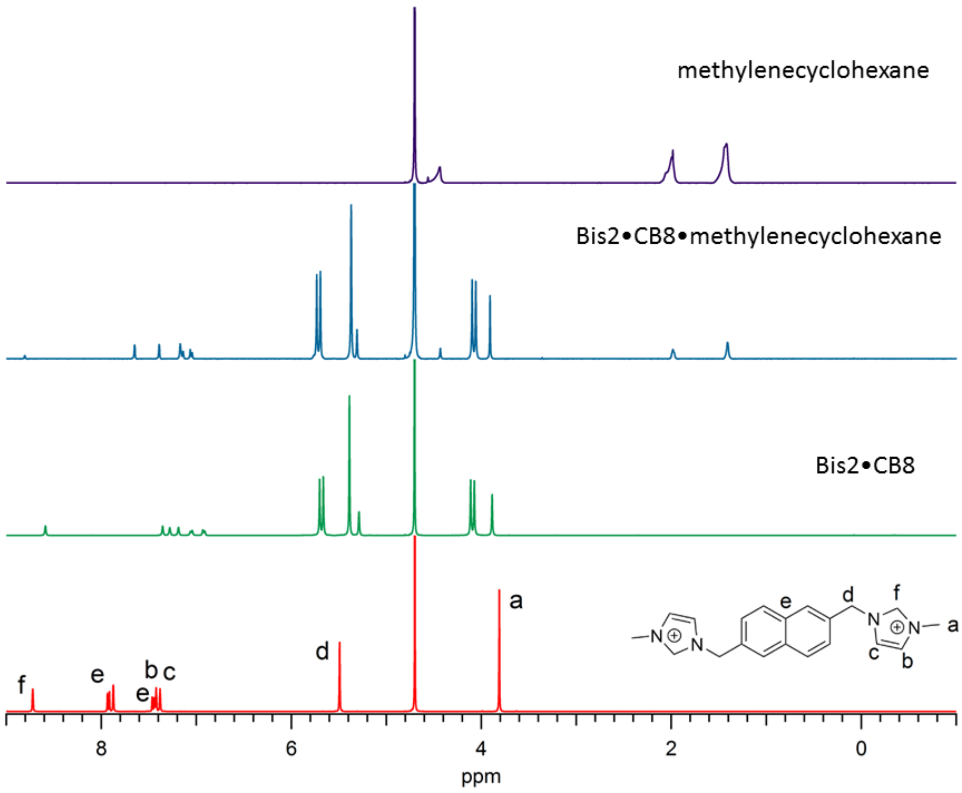

Figure S42. ^{1}H NMR spectra of bisimidazolium Bis2, Bis2•CB8 (1.25 mM), Bis2•CB8•methylenecyclohexane, and methylenecyclohexane free.

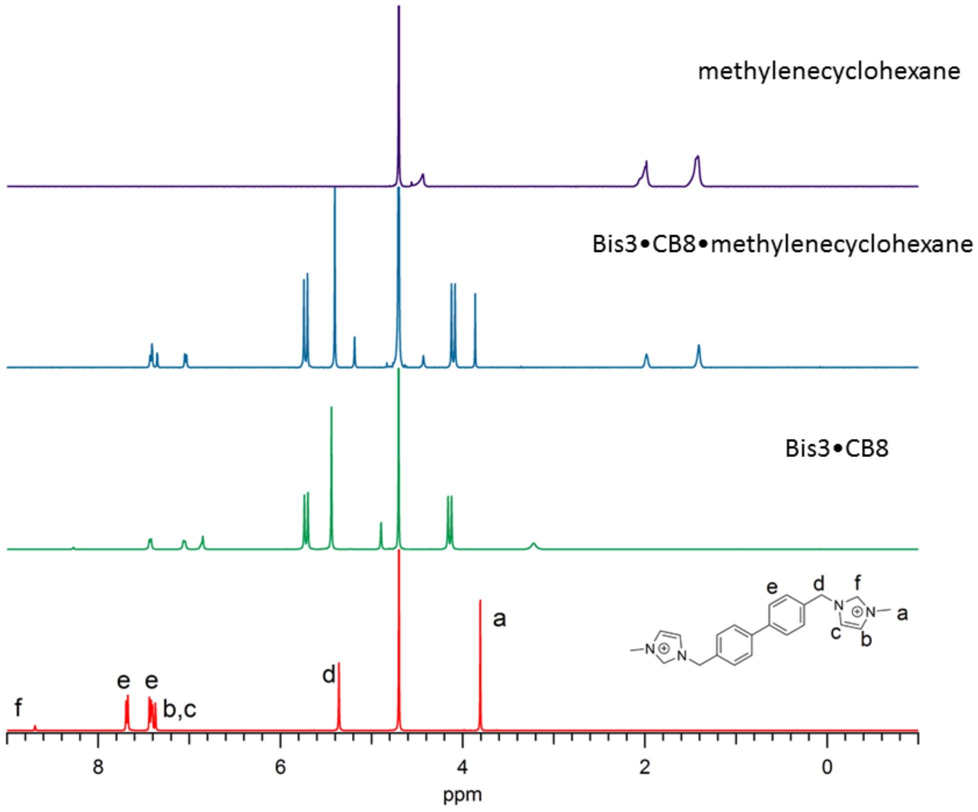

Figure S43. ^{1}H NMR spectra of bisimidazolium Bis3, Bis3•CB8 (1.25 mM), Bis3•CB8•methylenecyclohexane, and methylenecyclohexane free.

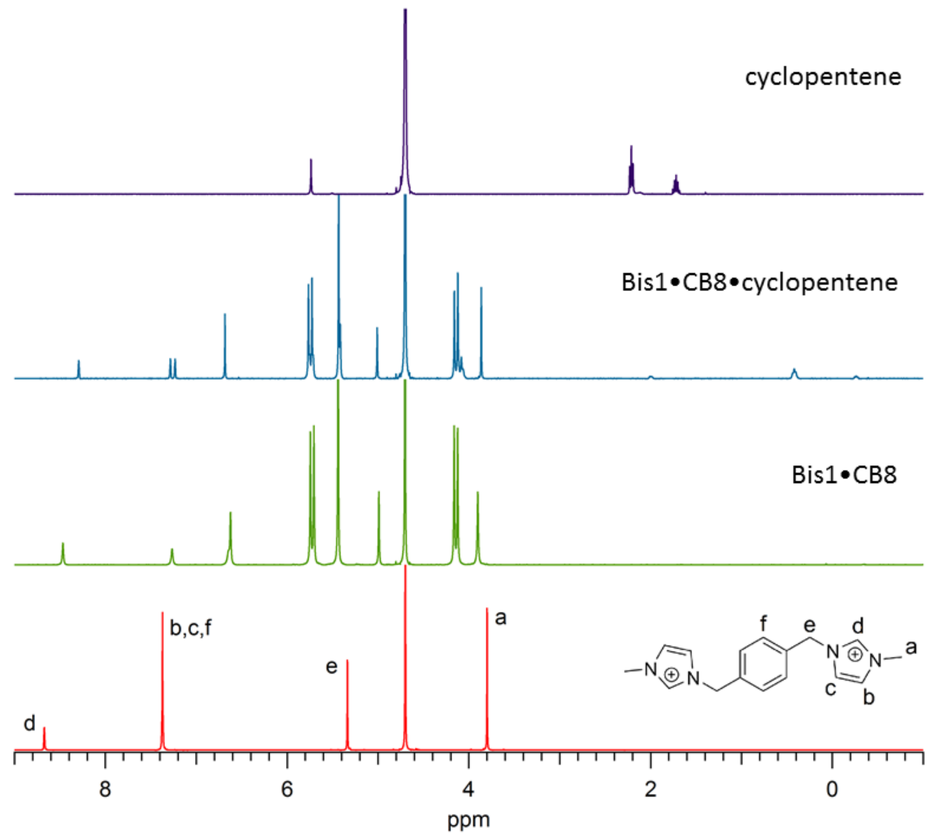

Figure S44. ^{1}H NMR spectra of bisimidazolium Bis1, Bis1•CB8 (1.25 mM), Bis1•CB8•cyclopentene, and cyclopentene free.

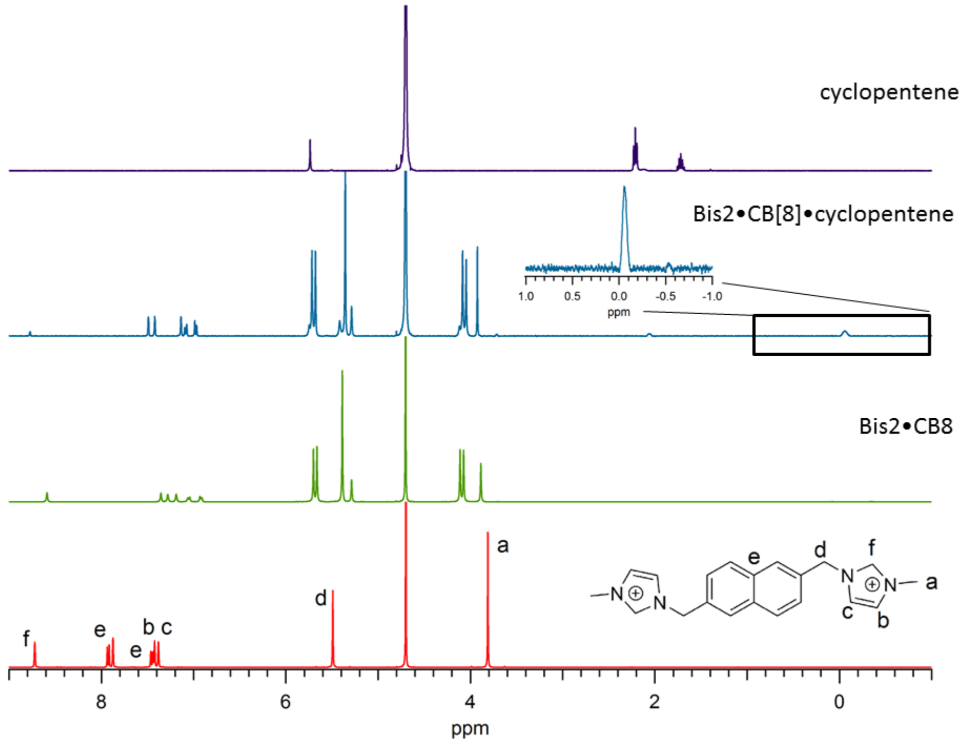

Figure S45. ^{1}H NMR spectra of bisimidazolium Bis2, Bis2•CB8 (1.25 mM), Bis2•CB8•cyclopentene, and cyclopentene free.

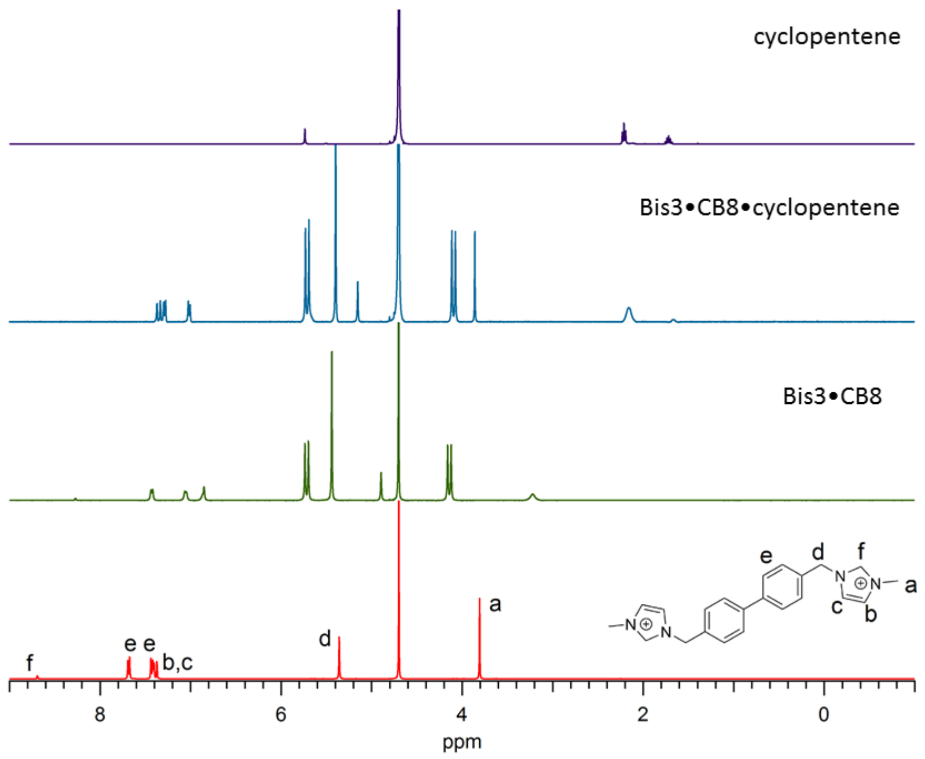

Figure S46. ^{1}H NMR spectra of bisimidazolium Bis3, Bis3•CB8 (1.25 mM), Bis3•CB8•cyclopentene, and cyclopentene free.

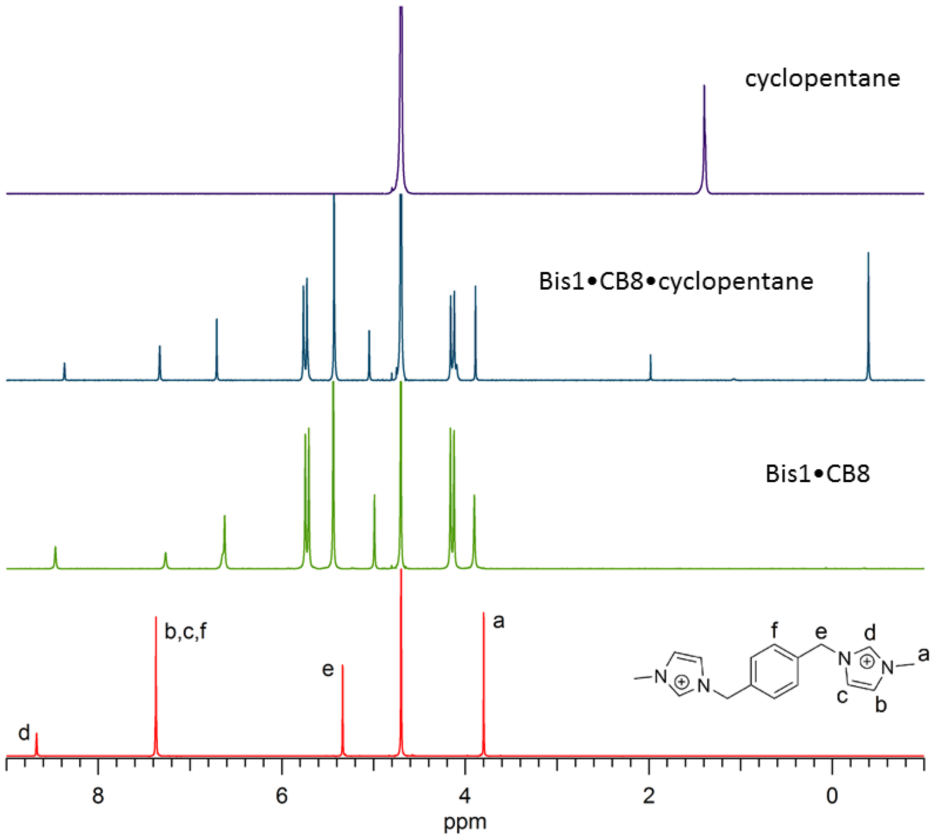

Figure S47. ^{1}H NMR spectra of bisimidazolium Bis1, Bis1•CB8 (1.25 mM), Bis1•CB8• cyclopentane, and cyclopentane free.

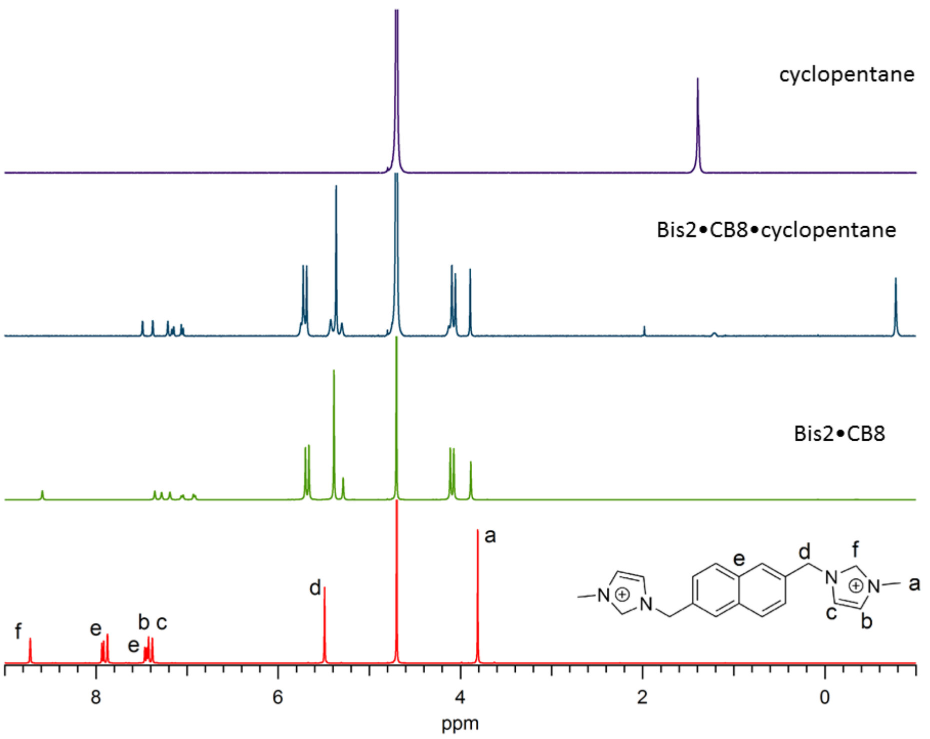

Figure S48. ^{1}H NMR spectra of bisimidazolium Bis2, Bis2•CB8 (1.25 mM), Bis2•CB8•cyclopentane, and cyclopentane free.

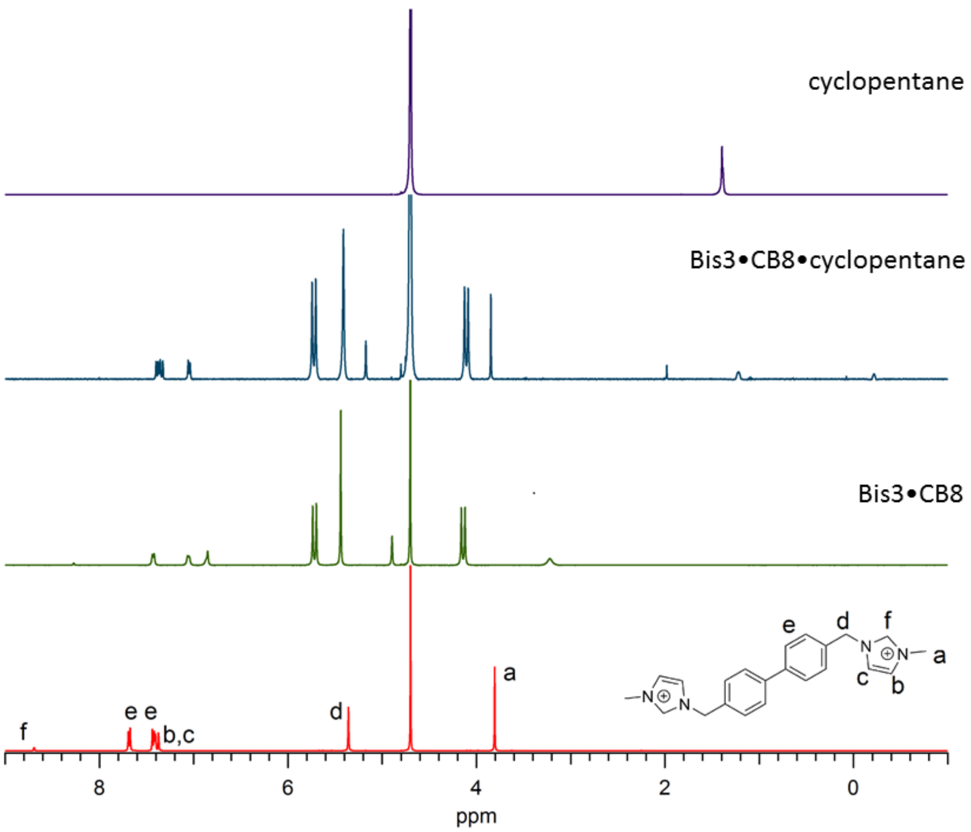

Figure S49. ^{1}H NMR spectra of bisimidazolium Bis3, Bis3•CB8 (1.25 mM), Bis3•CB8•cyclopentane, and cyclopentane free.

7. DFT Calculations

All DFT calculations were performed for the gas phase with the Gaussian software package.[1] The polarizabilities of Bis1-Bis3 were calculated with the B3LYP/aug-ccpvdz level of theory and the geometries of the binary complexes shown in Figure 1 at the wB97XD/3-21G* level of theory. In order to evaluate the preferred co-conformations of unsaturated guests inside the Bis2•CB8 complexes, we performed calculations for encapsulated *cis*-butene, isobutene, and benzene. The corresponding ternary complexes were geometry optimized with three different DFT methods (B3LYP, wB97XD, and M06-2X) by using the 6-31+G* basis set. The geometry optimizations were performed for two different co-conformations, one representing the presumed π-π interaction between the guest and the naphthalene ring of Bis2 ("stacked"), the other one corresponding to C–H---π interactions between the guest and the naphthalene π system ("orthogonal"), see representative optimized structures in Fig. S50. Note that in both conformations additional interactions with the two imidazolium groups apply, which cannot be readily dissected. The combined results, which revealed a clear preference (by 1-15 kcal mol^{-1}) for the stacked co-conformation for all three guests and at all three levels of theory in the gas phase, further support the interpretation of stabilizing π-π interactions between the naphthalene unit and the guests.

Table S5. DFT-calculated energies (in a.u.) and relative energies (in kcal mol^{-1}) for selected Bis2•CB8•guest complexes.[a]

guest	co-conformation	B3LYP	wB97XD	M06-2X
cis-butene	stacked (π–π)[b]	−5965.4412	−5963.8568	−5963.3268
	orthogonal (C–H---π)[c]	−5965.4377	−5963.8508	−5963.3252
	relative energy[d]	**−2.2**	**−3.8**	**−1.0**
isobutene	stacked (π–π)[b]	−5965.4423	−5963.8485	−5963.3248
	orthogonal (C–H---π)[c]	−5965.4274	−5963.8260	−5963.3138
	relative energy[d]	**−9.4**	**−14.1**	**−6.9**
benzene	stacked (π–π)[b]	−6040.4730	−6038.8672	−6038.3446
	orthogonal (C–H---π)[c]	−6040.4570	−6038.8496	−6038.3305
	relative energy[d]	**−10.0**	**−11.1**	**−8.9**

[a] 6-31+G* basis set. [b] The second guest is aligned to allow π-π interactions with the naphthalene unit, see Fig. S50. [c] The second guest is aligned to allow C-H---π interactions with the naphthalene unit, see Fig. S50. [d] Negative relative energy indicates a preference for the stacked (π-π) co-conformation.

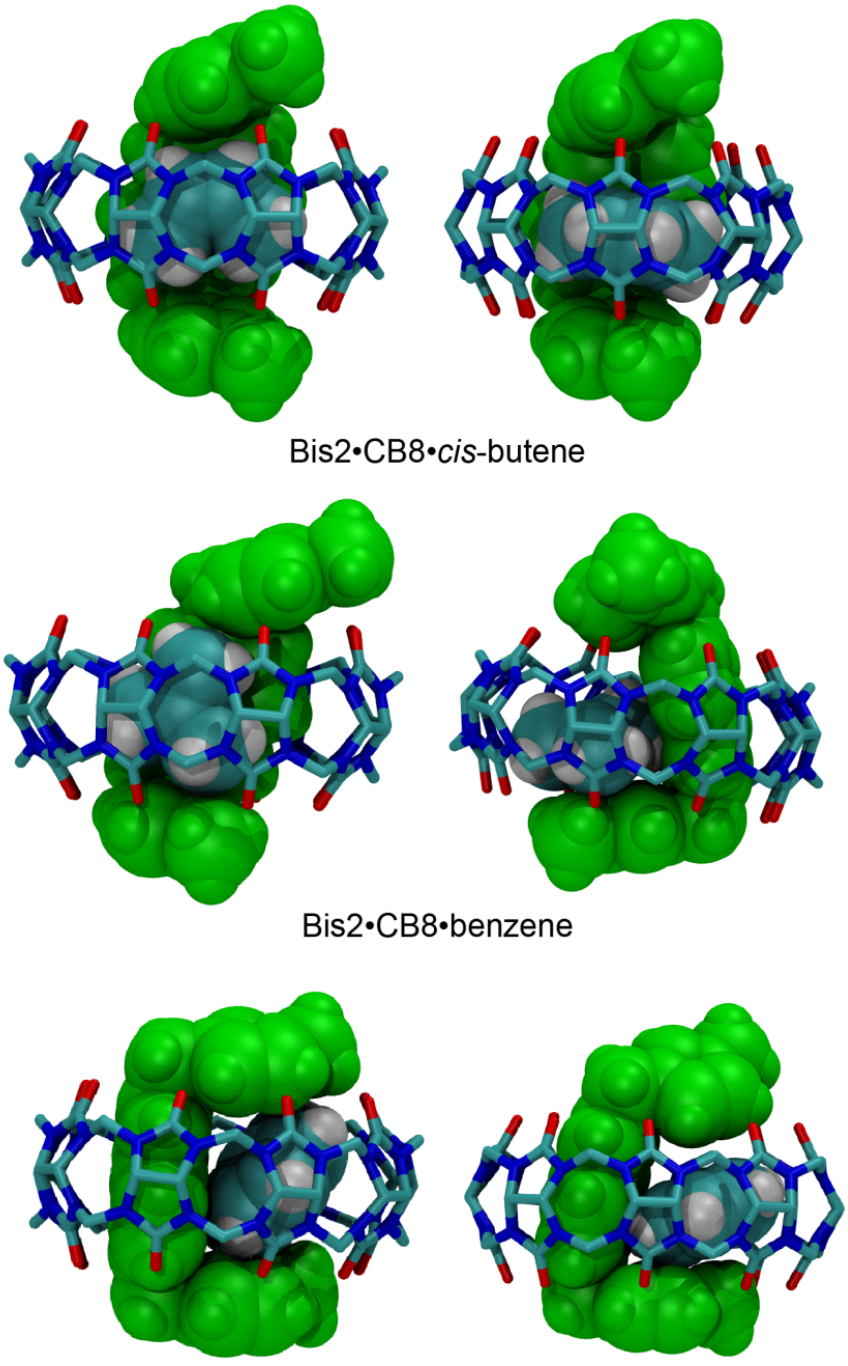

Figure S50. DFT-calculated structures (M06-2X/6-31+G*) of selected ternary Bis2•CB8•guest complexes. The structures on the left side represent optimized geometries corresponding to co-conformations that spatially allow π-π interactions with the naphthalene unit, the ones on the right to those that allow C-H---π interactions with the naphthalene unit.

8. References

1. M. J. Frisch, G. W. Trucks, H. B. Schlegel, G. E. Scuseria, M. A. Robb, J. R. Cheeseman, G. Scalmani, V. Barone, B. Mennucci, G. A. Petersson, H. Nakatsuji, M. Caricato, X. Li, H. P. Hratchian, A. F. Izmaylov, J. Bloino, G. Zheng, J. L. Sonnenberg, M. Hada, M. Ehara, K. Toyota, R. Fukuda, J. Hasegawa, M. Ishida, T. Nakajima, Y. Honda, O. Kitao, H. Nakai, T. Vreven, J. A. Montgomery, Jr., J. E. Peralta, F. Ogliaro, M. Bearpark, J. J. Heyd, E. Brothers, K. N. Kudin, V. N. Staroverov, T. Keith, R. Kobayashi, J. Normand, K. Raghavachari, A. Rendell, J. C. Burant, S. S. Iyengar, J. Tomasi, M. Cossi, N. Rega, J. M. Millam, M. Klene, J. E. Knox, J. B. Cross, V. Bakken, C. Adamo, J. Jaramillo, R. Gomperts, R. E. Stratmann, O. Yazyev, A. J. Austin, R. Cammi, C. Pomelli, J. W. Ochterski, R. L. Martin, K. Morokuma, V. G. Zakrzewski, G. A. Voth, P. Salvador, J. J. Dannenberg, S. Dapprich, A. D. Daniels, O. Farkas, J. B. Foresman, J. V. Ortiz, J. Cioslowski and D. J. Fox, *Gaussian 09*, Revision B.01, Gaussian, Inc., Wallingford CT, **2010**.
2. D. L. Mobley, Experimental and Calculated Small Molecule Hydration Free Energies. Department of Pharmaceutical Sciences, UCI, **2013**. http://escholarship.org/uc/item/6sd403pz/ (accessed October 14, 2019).
